# Aroma characterization based on aromatic series analysis in table grapes

**DOI:** 10.1038/srep31116

**Published:** 2016-08-04

**Authors:** Yusen Wu, Shuyan Duan, Liping Zhao, Zhen Gao, Meng Luo, Shiren Song, Wenping Xu, Caixi Zhang, Chao Ma, Shiping Wang

**Affiliations:** 1 Department of Plant Science, School of Agriculture and Biology, Shanghai Jiao Tong University, Shanghai, China

## Abstract

Aroma is an important part of quality in table grape, but the key aroma compounds and the aroma series of table grapes remains unknown. In this paper, we identified 67 aroma compounds in 20 table grape cultivars; 20 in pulp and 23 in skin were active compounds. C_6_ compounds were the basic background volatiles, but the aroma contents of pulp juice and skin depended mainly on the levels of esters and terpenes, respectively. Most obviously, ‘Kyoho’ grapevine series showed high contents of esters in pulp, while Muscat/floral cultivars showed abundant monoterpenes in skin. For the aroma series, table grapes were characterized mainly by herbaceous, floral, balsamic, sweet and fruity series. The simple and visualizable aroma profiles were established using aroma fingerprints based on the aromatic series. Hierarchical cluster analysis (HCA) and principal component analysis (PCA) showed that the aroma profiles of pulp juice, skin and whole berries could be classified into 5, 3, and 5 groups, respectively. Combined with sensory evaluation, we could conclude that fatty and balsamic series were the preferred aromatic series, and the contents of their contributors (β-ionone and octanal) may be useful as indicators for the improvement of breeding and cultivation measures for table grapes.

Aroma is an important aspect of quality in grapes and wines and, hence, for consumer acceptance^1^,^2^,^3^,^4^,^5^,^6^,^7^,^8^. Several families of compounds are responsible for the aroma of grapes. Among them, esters and terpenes are known to contribute to fruity/floral characters^9^,^10^; C_6_-aldehydes and alcohols possess green leafy aroma characters^11^; methoxypyrazines are strongly linked to green capsicum descriptors^8^,^12^. Meanwhile, C_13_-norisoprenoids generally contribute to many flavours^13^,^14^ in fruits and wines, such as berry, tobacco, honey, balsamic and violet aromas.

Aroma compounds are usually located in both pulp and skin as free and bound glycosides^1^. Bound glycoside forms, which can be transformed by hydrolysis into odour-active forms (free fractions), increasing the aromatic characteristics of the grape, are non-volatile compounds with no direct contribution to the aroma of the grape^15^. In contrast, free forms are volatile compounds that can be sensed and tasted directly and are involved in the aroma of grape berries. Obviously, free forms are vital ingredients and completely determine the flavour of table grapes. However, not all volatiles contribute with the same intensity to aroma. The concentration-odour threshold ratio, known as the “odour activity value” (OAV), must be considered as the only principle to estimate the contribution of each compound to aroma, although interactions (antagonistic and additive effects) among different aroma components occur in the matrix^9^,^12^. Because an individual compound generally has several flavours^16^, it is difficult to establish or evaluate global aroma profiles only using the odour activity values (OAVs) of volatiles. Grouping the OAVs of the aroma compounds with similar descriptors into aromatic series makes up the organoleptic profiles (OAV Aroma Wheel). This process relates quantitative information acquired by chemical analysis to sensory perceptions^12^ and thus simply and effectively evaluates and compares the aroma characters^9^. This method has recently been employed to distinguish wine grape varieties^4^,^12^,^17^ and wines^9^,^16^.

Currently, most of the studies on aroma have focused on wine grapes and wines and have made noticeable progress in many aspects, including varieties^12^, regions^18^, training systems^19^, developmental stages^11^,^20^, aromatic maturity^21^ and positions of berries^4^,^17^. Regarding table grapes, Yang *et al*. evaluated the volatile compounds in certain grapes at the germplasm level^22^ and optimized the harvest dates of three grapes^23^; Fenoll *et al*. predicted the Muscat aroma in table grapes via analysis of rose oxide^24^ and determined the evolution of aromatic compounds in Muscat Hamburg during ripening^10^. There remains a lack of research on the aspects of aromatic series and aroma profiles in table grapes, as well as a lack of effective indicators for evaluating or determining the aroma quality.

Table grapes account for 80% of grape production in China^22^. It is very important to fill the research gaps in the evaluation system for table grape aroma qualities. In the present study, the 20 most popular table grape cultivars were selected for determination and analysis of the free aroma compounds in both skin and pulp juice by automatic solid-phase microextraction (SPME) combined with gas chromatography-mass spectrometry (GC-MS). Then, the aromatic series and aroma profiles of table grapes were constructed, and their similarity or dissimilarity was revealed by multivariate data analysis. Furthermore, the preferred series and their corresponding aroma components were confirmed by an additional sensory evaluation, which can be used as an important indicator for the improvement of the breeding and cultivation measures of table grapes. Finally, the aroma characters among table grapes, wine grapes and wine were compared.

## Results

### Determination of table grape maturity

**Figure 1:**
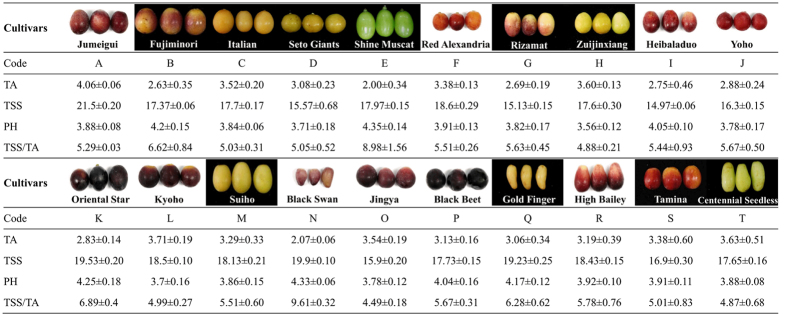
The 20 main table grape cultivars used in this work and their total acidity (TA, g/L), total soluble solids (TSS, °Brix), pH and sugar:acid ratio (TSS/TA). Data are shown as the means ± standard error (n = 3). The photographs of grape cultivars at their harvest date are provided on the cultivar rows. Capital letters indicate the codes of the table grape cultivars: A-Jumeigui, B-Fujiminori, C-Italian, D-Seto Giants, E-Shine Muscat, F-Red Alexandria, G-Rizamat, H-Zuijinxiang, I-Heibaladuo, J-Yoho, K-Oriental Star, L-Kyoho, M-Suiho, N-Black Swan, O-Jingya, P-Black Beet, Q-Gold Finger, R-High Bailey, S-Tamina, and T-Centennial Seedless.

To estimate the maturity of 20 table grape cultivars, the total soluble solids (TSS), the total acidity (TA), the sugar:acid ratio (TSS/TA) and the pH were identified (Fig. 1). In these cultivars, the TA ranged from 2.50–4.00 g/L, and TSS values were higher than 15 °Brix in almost all cultivars. The TSS/TA ranged from approximately 5 to 7, except for those of Shine Muscat (8.98) and Black Swan (9.61). These results indicated that all of the grape berries reached their appropriate maturity times.

### Identification and characterization of the volatile compounds in table grapes

**Figure 2:**
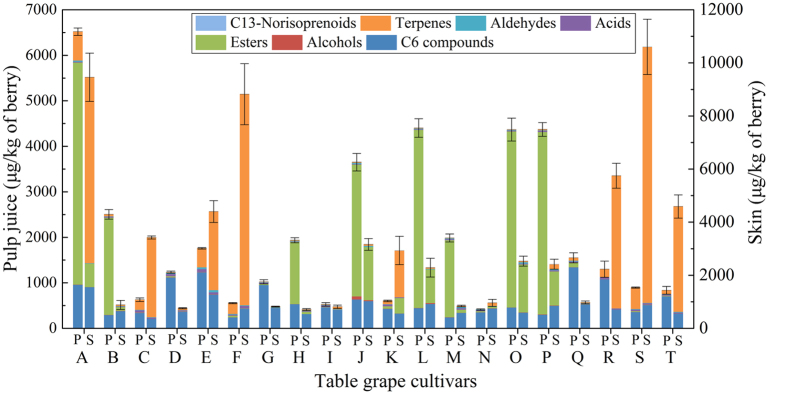
Each class of volatile compounds as measured in the pulp juice (P) and skin (S) of table grapes. The results are shown as the mean values. The error bars represent the standard deviation (n = 3). Capital letters refer to the table grape cultivars as listed in Fig. 1.

A total of 67 volatile compounds, 61 in pulp (Table 1) and 64 in skin (Table 2), were identified in these 20 cultivars, and these compounds included 8 alcohols, 24 terpenes and 12 esters in skin and 6 alcohols, 21 terpenes and 14 esters in pulp juice. The other 3 acids, 7 aldehydes, 3 C_13_-norisoprenoids and 7 C_6_ compounds were found in both skin and pulp juice. The total aroma contents exhibited significant differences between skin and pulp juice from the 20 grape cultivars (Fig. 2). Esters were primarily located in pulp, whereas terpenes were mostly found in skin. Regarding the total contents of volatiles, due to the high level of esters, pulp juice was richer than skin in ten cultivars (e.g., Seto Giants, D), whereas for the remaining cultivars, pulp juice showed lower levels than skin because of the high levels of terpenes in the skin.

In pulp juice (Fig. 2), the total aroma contents in eight cultivars (A (maximum, 6519.54 μg/kg), B, H, J, L, M, O, P) were significantly higher than in the other cultivars. In these eight cultivars, esters were the predominant volatile compounds (70.60–91.69%), and ethyl acetate accounted for 96.68–99.72% of the total concentrations of esters (Table 1). In skin (Fig. 2, Table 2), terpenes were the predominant volatile compounds (60.01–90.97%) in another eight cultivars (A, C, E, F, K, R, S, T), in which the total contents exceeded 2.90 mg/kg. Monoterpenes were the dominant terpenes, and in most cultivars, acyclic monoterpenes were the major monoterpenes (Supplementary Tables S1 and S2) due to their synthetic pathway^25^. However, cyclic monoterpenes, chiefly D-Limonene, were the dominant monoterpenes identified from the pulp juice of B, M, N, Q or the skin of D, H, N, Q. Esters were also present in the skin of four cultivars (L, O, P, J) as dominant volatiles due to the high level of ethyl acetate but occurred at much lower levels than those in pulp juice (Fig. 2). C_6_ compounds were present in both pulp juice and skin, with nonnegligible concentrations (ranging from 240.81–1552.20 μg/kg) in all samples (Fig. 2, Tables 1 and 2), and they represented the main volatiles identified in the pulp juice of ten cultivars (e.g., Fujiminori, B) and the skin of eight cultivars (e.g., Yoho, J). Hexanal, (***E***)-2-hexenal, hexanol and (***E***)-2-hexenol were the dominant C_6_ volatile compounds (Tables 1 and 2). Among them, the concentrations of (***E***)-2-hexenal and (***E***)-2-hexenol were higher than those of hexanal and hexanol in the berries of most cultivars, except for the pulp juice of four samples (C, G, N, R) and the skin of four samples (D, N, E, K), in which the (***E***)-2-hexenol concentration was lower than that of hexanol. In addition, the (***Z***)-3-hexenol concentration was relatively high in Shine Muscat (E) berries (Tables 1 and 2). In conclusion, C_6_ volatile compounds were the basic background volatiles of table grape berries, and the total contents of volatiles in pulp and skin largely depended on the levels of esters and terpenes, respectively.

Grape berry volatiles were synthesized mainly from fatty acids (straight-chain compounds), amino acids (branched compounds) and isoprenoids (terpenes and C_13_ compounds)^26^. In this work, straight-chain aroma compounds were the predominant components in all grape pulp juice and most skin (Supplementary Tables S3 and S4), while terpenes were the main aroma compounds in the skin of eight cultivars (e.g., Tamina, S), which suggested that volatiles in table grape berries (skin and pulp) were derived primarily from fatty acids, except in the skins of eight cultivars, in which aroma compounds were synthesized via the isoprenoid pathway.

### Active odorants

Numerous aroma compounds are present in fruits, and flavour intensity depends on both concentration and threshold. However, only a limited number of volatiles can be found at concentrations high enough to be perceived (OAV ≥ 1) and considered as flavour contributors as well as active odorants^12^. Therefore, only a few of the active odorants are primarily responsible for the fruit aroma^10^,^27^. For example, in 10 litchi varieties^28^, only 4 monoterpenes were active odorants throughout the maturation period^10^, and 13 odorants were deemed to contribute to overall profiles, whereas 96 volatiles were detected.

A total of 20 and 23 aroma-active compounds (OAVs > 1) were detected in the pulp and skin of 20 table grape cultivars, respectively (Tables 3 and 4). C_6_, terpenes and C_13_-norisoprenoid compounds were the main contributors to aroma, and their OAVs were higher in skin than in pulp juice. Hexenal and (***E***)-2-hexenal were the most active C_6_ compounds, and they contributed to the flavours of table grape samples, except for the pulp of Black Beet (P). The OAVs of C_6_ compounds obtained from the pulp juice of Jumeigui (A), High Bailey (R), and Rizamat (G) and from the skin of Jumeigui (A), Shine Muscat (E), and Tamina (S) were significantly higher than those in other cultivars (OAVs of C_6_ compounds >90). In addition, (***Z***)-3-hexenal and (***Z***)-3-hexenol could also provide effective aroma from the skin or pulp of some cultivars at low intensity (OAVs < 5). The aroma intensities (>100) of terpenes in the berries of seven cultivars (A, C, E, F, R, S, T) were higher than those in other cultivars. In pulp juice, linalool was the most active odorant, whereas the skin aroma was derived primarily from geranic acid, geraniol and linalool. Traces of rose oxides were detected in some grape berries, but these compounds only exceeded their odour thresholds in Jumeigui (A) and Shine Muscat (E). Moreover, the aroma contributed by terpenes was weak (OAVs < 20) in twelve cultivars (e.g., Fujiminori, B). Although present at low concentrations, C_13_-norisoprenoids, particularly β-damascenone and β-ionone, made significant contributions to the aroma of grape berries due to their low thresholds (Table 5). Except for the pulp juice of four cultivars (B, C, K, P), both β-damascenone and β-ionone may contribute to the powerful aroma of all grape samples. Among them, the OAV for β-ionone in Shine Muscat (E) berry was significantly higher than in other cultivars. Regarding alcohols, aldehydes and acids, these compounds exhibited relatively little odour because of their high thresholds (Table 5). For example, 1-octen-3-ol provided a slight smell of mushroom or earth in the pulp of two cultivars (A, J) and the skin of four cultivars (J, O, P, L), and ethyl butyrate, with slightly higher OAVs, contributed fruity flavour to the pulp of six cultivars (e.g., Black Beet, P).

### Aromatic series

**Figure 3:**
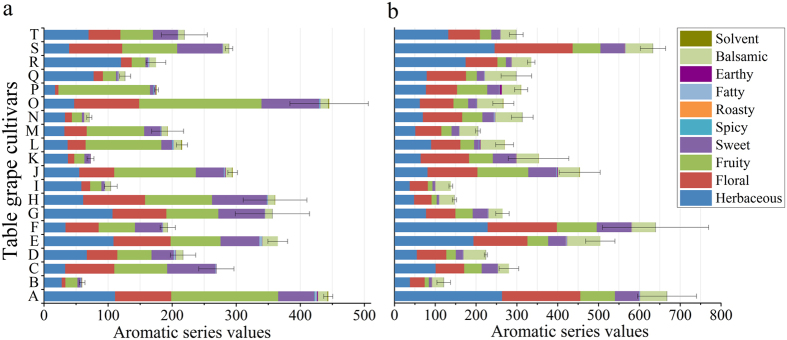
Aromatic series values for pulp juice (**a**) and skin (**b**) of table grapes. The results are shown as the mean values. The error bars represent the standard deviation (n = 3). Capital letters refer to the table grape cultivars as listed in Fig. 1.

Calculation of the aroma series by the accumulation of OAVs cannot be interpreted as an arithmetical addition of odorant sensations, and the assignment of some compounds in a particular series or in several series may be arguable^17^,^29^,^30^. However, the proposed method, which groups the compounds into odorant series, reduces the number of variables to be interpreted and, consequently, is valid and simply for comparing the fruits’ aroma characters. Remarkably, some compounds have a dual sensory effect^9^,^21^,^26^,^31^: a positive sensory effect was attributed to low concentration, and a negative sensory effect was attributed to increased concentrations. In this study, these compounds were present at low levels^9^,^21^,^26^,^31^ and showed corresponding positive sensory attributes, as listed in Table 5. Accordingly, ten aromatic series of 20 table grape cultivars based on OAVs were established, as shown in Fig. 3. Among them, grape berries were characterized mainly by herbaceous, fruity, sweet, floral and balsamic series, but the values behaved significantly differently in different grape cultivars and different berry parts. The pulp juice (Fig. 3a) of A and O (>400) and the skin (Fig. 3b) of A, F, and S (>600) showed relatively high levels of aromatic series, which suggested that these grape parts provided more powerful aroma than the other parts. Meanwhile, the pulp juice of B, K, and N (<100) and the skin of B, H and I (<200) displayed weak aroma.

The herbaceous series showed a higher intensity in pulp juice (>100) from A, E, G, and R and in skin (>200) from A, F and S; the floral series showed a higher level in pulp juice (>80) from six cultivars (O, H, S, E, G, A) and in skin (>160) from A, F and S; sweet aroma was rich (>70) in pulp juice from five cultivars (O, H, C, G, S) and Red Alexandria (F) skin; meanwhile, the pulp juice from six cultivars (A, J, M, L, O, P) and the skin of Yoho (J) showed high levels (>100) of fruity flavour. Regarding the balsamic series, the maximum value was found in Shine Muscat (E) pulp (23.87) and skin (80.89), and the former also showed the highest content of fatty flavour (4.28). In skin, Black Swan (N) showed the highest fatty flavour (2.51), followed by Shine Muscat (E) (2.39). The pulp from A and J and skin from P, J and L presented slight earthy flavour. Only A, O and L pulp showed solvent aroma, but the values (<1.2) were low. In addition, all grape samples, both pulp and skin, showed no odour value for spicy and roasty (<1), which suggested that these types of flavours cannot be perceived by humans.

**Figure 4:**
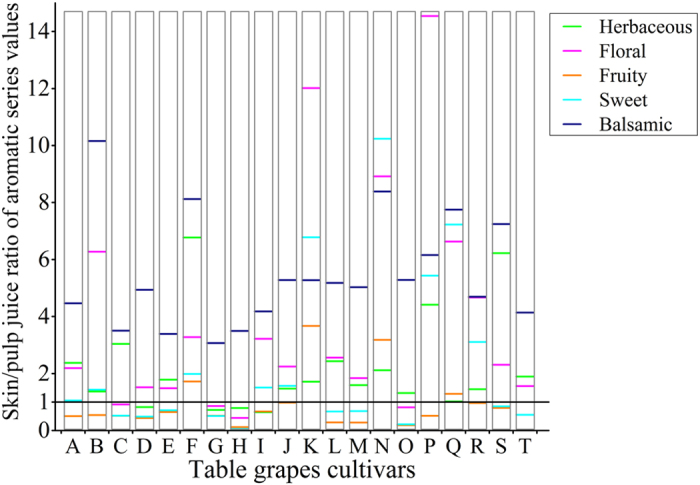
Skin/pulp juice ratio of aromatic series values in five main aroma series. Data are the means (n = 3). Capital letters refer to the table grape cultivars as listed in Fig. 1.

In most cultivars, the skin/pulp juice ratios for the five primary aroma series showed higher values of herbaceous, floral, and balsamic series in skin than in pulp (ratios > 1) but higher values of fruity series in the pulp juice (ratios < 1) (Fig. 4). This finding suggests that table grape pulp has a stronger fruity aroma and that the skin presented more a herbaceous, floral, balsamic aroma. However, in five cultivars (F, K, N, Q), the five major aroma series were all higher in skin than in pulp, which differentiated these cultivars from the others. For the sweet series, the skin of ten cultivars (e.g., Jumeigui, A) showed higher values than those in pulp, with the maximum ratio (10.24) observed in Black Swan (N) and the minimum ratio (0.06) observed in Zuijinxiang, H.

### Aroma fingerprints

To simply and effectively show the aroma profiles of table grapes, aroma fingerprints were established using an OAV Aroma Wheel (Supplementary Fig. S1). For example, the visual aroma fingerprints showed that herbaceous was the predominant flavour in High Bailey, that fruity flavour was abundant in Black Beet, and that Jumeigui, Shine Muscat, Red Alexandria and Damina showed high abundances of herbaceous, fruity, sweet, and floral series.

### Multivariate data analysis

Hierarchical cluster analysis (HCA) is suitable for classifying samples into different classes or clusters. Samples in the same cluster display similar profiles, whereas samples in different clusters are obviously different^32^. Principal component analysis (PCA) is a multivariate data analysis technique used for dimensionality reduction and for showing relationships/correlations between the variables and samples^32^,^33^. Both of these approaches have been widely used in the aroma study of grape and wine^9^,^15^. In this study, OAVs of 6 aromatic series, the 5 primary series and fatty aroma (abundant in some cultivars) were used as the variables. On the basis of aroma profiles, the 20 table grape cultivars were divided into several groups, and the characters of each group were analysed using the above-mentioned statistical methods.

**Figure 5:**
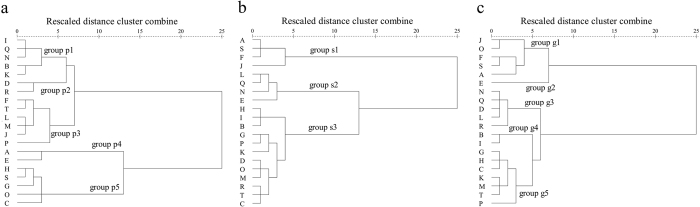
Dendrograms for the hierarchical cluster analysis (HCA) results using Ward’s cluster algorithm for the dataset of six aromatic series obtained from table grapes. (**a**) Pulp juice samples were divided into five clusters: groups p1, p2, p3, p4, and p5. (**b**) Skin samples were divided into three clusters: groups s1, s2, and s3. (**c**) Whole grape berry samples were divided into five clusters: groups g1, g2, g3, g4, and g5.

**Figure 6:**
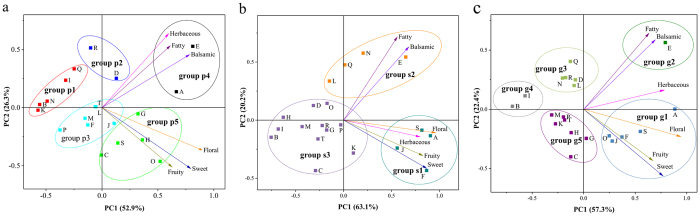
Biplots (score plots combined with loading plots) of principal component analysis (PCA) results based on the six aromatic series obtained from table grapes. (**a**) Pulp juice. (**b**) Skin. (**c**) Whole grape berries.

The results of multivariate data analyses of 20 table grape cultivars are shown in Figs 5 and 6. The aroma profiles of pulp juice, skin and whole grape berries were appropriately divided into five (Fig. 5a), three (Fig. 5b) and five (Fig. 5c) clusters, respectively, by hierarchical cluster analysis. The PC1 (first principal component) and the PC2 (second principal component) accounted for 79.20% (Fig. 6a), 83.3% (Fig. 6b) and 79.7% (Fig. 6c) of the total variance in pulp juice, skin and grape berries, respectively. This finding indicated that these factors were considered sufficient for further discussion. Grape cultivars were also clearly separated by two PCs. This result was in agreement with the HCA (Fig. 5) and thus further validated the objectivity and rationality of the data analysis.

Biplots (score plots combined with loading plots, Fig. 6) of the PCA showed that the 6 aromatic series were scattered in quadrants I and IV, indicating their positive correlations with PC1, which explained more than 50% of the total variability of each series. Among the 13 total groups, the cultivars from groups p1, s3 and g4, with negative values for PC1, had lower values for the 6 aromatic series than did the other cultivars.

Groups p3 and g5 were projected near the origin and had small negative values for PC2, which revealed that the cultivars of the former were low in herbaceous but slightly rich in fruity and that the latter were somewhat rich in floral, fruity and sweet. However, the cultivars of groups p2, s2 and g3 had positive values for PC2. Based on this result, the pulp juices of Seto Giants (D) and High Bailey (R) (group p2) showed lower values in floral and sweet but slight richness in herbaceous components; group s2, especially Shine Muscat (E), together with group g3, exhibited slight richness in the fatty and balsamic series.

The remaining 5 groups (group p4, p5, s1, g1, g2) were in the positive region of PC1, indicating that the values of the 6 aromatic series were significantly higher in these cultivars than in the other cultivars. Samples from group s1 (A, S, J, F) were significantly higher in herbaceous, fruity, sweet, and floral series than the other cultivars; groups p4 and g2 (pulp of Shine Muscat (E) and Jumeigui (A) and the grape berries of Shine Muscat (E)) exhibited the highest values for the fatty and balsamic series, as well as moderate richness in herbaceous aroma. Meanwhile, groups p5 and g1 were located in quadrant IV, indicating that these samples had higher values in the sweet, floral and fruity series.

Among the groups falling in quadrants I and IV, we noted that the pulp, skin and berries of Shine Muscat (E) were discriminated from the other cultivars due to their high values for the fatty and balsamic series. In addition, Fig. 6 shows the highly positive correlations that were found between the fatty and balsamic series and that positive correlations were observed among floral, fruity and sweet series in both the pulp and skin. Meanwhile, herbaceous aroma was positively correlated with fatty and balsamic series in pulp juice (Fig. 6a) but with floral, fruity and sweet series in skin (Fig. 6b).

## Discussion

Most table grapes belong to *V. vinifera* and its hybrids (*V. vinifera* × *labrusca*). The volatiles of berries are synthesized primarily via the fatty acid, amino acid and isoprenoid pathway^26^. In comparison to *V. vinifera*, the hybrids show high levels of esters, among which ethyl acetate is the most important compound^22^. According to the analysis of aroma components in both pulp and skin (Fig. 2, Tables 1 and 2), we found that ethyl acetate (96.68–99.72% of total esters) was the primary compound in the pulp of the eight hybrids (A, B, H, J, L, M, O, P) that had the highest levels of pulp aroma compounds (esters accounted for 70.60–91.69%). It is interesting that Shine Muscat (E), Gold Finger (Q) and Oriental Star (K) showed low levels of esters despite also being hybrids. The presence of esters is caused mainly by β-oxidation enzyme activity and specificity in the fatty acid metabolism pathway^34^. Notably, the above-mentioned eight cultivars (A, B, H, J, L, M, O, P) are Kyoho grapevine series (Kyoho or its offspring, Fig. S2), which have a number of advantages, such as large berries, beautiful colour, and disease resistance, and these cultivars occupy the largest planting area in China. In addition, esters were also detected in the skin of four cultivars (J, L, O, P) of Kyoho grapevine series but at a lower level than in pulp (Fig. 2). Therefore, the main aroma characteristic of Kyoho grapevine series is that the pulp aroma components are provided mainly by ethyl acetate, which was mostly inherited from their parent V. labrusca^22^. However, because of the high odour threshold (Table 5), ethyl acetate cannot provide effective aroma to Kyoho grapevine series (Table 3). Consequently, their flavours were produced primarily by other active compounds, which were observable in the aromatic series (Fig. 3a).

Terpenes, synthesized from glucose via the isoprenoid pathway, are abundant compounds in both Muscat and non-Muscat aromatic varieties and provide significantly floral and fruity aroma to berries^35^. Based on the levels of free monoterpenes in pulp juice, *V. vinifera* wine grapes could be grouped into Muscat/floral cultivars (≥6 mg/L), non-Muscat aromatic cultivars (1–4 mg/L) and neutral cultivars (<1 mg/L)^36^. The *V. vinifera* table grapes have long lacked a standardized method of aroma analysis. Unlike wine grapes, which show abundant free monoterpenes in pulp, table grapes are rich in free monoterpenes in the skin (Table 2, Supplementary Table S2). Therefore, we established the aroma classification of table grapes (*V. vinifera*) based on contents of skin monoterpenes by imitating the classification of wine grapes. According to this classification, Red Alexandria (7.95 mg/kg), High Bailey (5.00 mg/kg) and Damina (9.64 mg/kg) (F, R, S) fell into the category of Muscat/floral cultivars, whereas Italian (3.00 mg/kg) and Centennial Seedless (3.97 mg/kg) (C, T) were placed in the non-Muscat aromatic cultivars (Supplementary Table S2). Because the V. vinifera parent species contain Muscat aroma^22^, abundant monoterpenes were also detected in the hybrids (*V. vinifera* × *V. labrusca*) Jumeigui (7.00 mg/kg), Shine Muscat (2.95 mg/kg) and Oriental Star (1.76 mg/kg) (A, E, K). The other grapes tested were neutral cultivars (<1 mg/L). In addition, based on their mechanisms of synthesis (Supplementary Fig. S3), monoterpenes have been divided into acyclic and cyclic compounds^25^, with the former being the predominant monoterpenes in grape berries^10^,^35^. Similarly, in this study, monoterpenes were mostly present in the form of acyclic components in both pulp and skin. However, cyclic monoterpenes were dominant compounds in the pulp (B, M, N, Q) and skin (D, H, N, Q) of four cultivars each (Supplementary Tables S1 and S2), which indicated that monoterpenes were directly synthesized from α-terpinyl cation in these tissues^25^ (Supplementary Fig. S3).

C_6_ compounds, derived from unsaturated fatty acids (linolenic and linoleic acid)^20^ (Supplementary Fig. S4), formed one of the most important aroma classes in grape berries. Upon analysis of C_6_ compounds in wine grapes, the linolenic acid pathway (route A) predominated over the linoleic acid pathway throughout berry development, whereas the linoleic acid pathway was more dominant after veraison^11^,^20^. In contrast to wine grapes, in most of the table grape cultivars, (***E***)-2-hexenal and (***E***)-2-hexenol were richer than hexanal and hexanol (Tables 1 and 2), which indicated that the linolenic acid pathway (route A) was the primary metabolic pathway due to differences in alcohol dehydrogenase (ADH) activity and substrate preference^11^. However, the linoleic acid pathway predominated over the linolenic acid pathway in the pulps (C, G, N, R) and skins (D, E, K, N) of four cultivars each, in which the level of (***E***)-2-hexenol was lower than that of hexenol. In addition, the linolenic acid pathway (route B) was also active in Shine Muscat (E) berries based on its high level of (***Z***)-3-hexenol.

Active odorants are the direct components of aromatic series. The specific intensity and tendency of active odorants could be obtained by combining OAVs and odour descriptors. In table grapes (Tables 3 and 4), (***E***)-2-hexenal and hexenal were the primary contributors to herbaceous aroma (Table 5); floral aroma was produced by terpenes (including rose oxides, linalool, geraniol, citronellol and nerol) and C_13_-norisoprenoids (including β-damascenone and β-ionone). Among them, linalool, β-damascenone and β-ionone were the primary contributors. Meanwhile rose oxides, with a rose note^37^, were active components only in Jumeigui (A) and Shine Muscat (E) and, consequently, could be used as the signature aroma. Recent studies reported that rose oxide was above the odour threshold (OAV > 1) in all of the Muscat (e.g., Muscat of Alexandria) and slight-Muscat cultivars but was absent in non-Muscat cultivars^24^. However, an early study reported that rose oxide was not detected in Muscat of Alexandria^35^. The above results indicated that terpenes were also influenced by many factors, such as terroir and climate, in addition to cultivar. Contrary to the results reported by Ruiz-García *et al*., in the present study, rose oxides were detected in Muscat and slight-Muscat cultivars, such as Red Alexandria (F), but their concentrations were below the threshold (0.5 μg/L), except in Jumeigui (A) and Shine Muscat (E). In addition to floral aroma, linalool and β-damascenone also made considerable contributions to sweet^14^,^16^,^38^,^39^,^40^,^41^ and fruity flavour^35^. For example, β-damascenone can provide baked apple flavour^42^. Esters, especially ethyl butyrate and ethyl hexanoate, were typically responsible for fruity aroma. In addition, fruity aroma could also be strengthened in some grape samples by octanal^42^,^43^, nonanal^43^ and limonene^44^ due to their lemon flavour. The fatty and balsamic series were contributed mainly by octanal and β-ionone, respectively, and in particular, the latter could show strong balsamic aroma, such as in Shine Muscat (E), because of its low threshold (0.007 μg/l) in water. 1-Octen-3-ol was the only contributor of earthy aroma in table grape^38^,^45^.

To our knowledge, for table grapes, there is not only a lack of studies evaluating aromatic series and aroma profiles but also no simple, pictorial, and comprehensive indicator for understanding the aroma characters. To address these issues, we established the aroma fingerprints as a visual radar chart (OAV Aroma Wheel) (Supplementary Fig. S1). This approach not only establishes the aroma files^9^ and consequently allows the extraction of useful aroma information but also provides a simple and effective basic indicator of aromas for ordinary people. With the development of cultivation techniques, increasing numbers of table grapes are being vacuum-packed in plastic packages before being sold in markets in China. Therefore, like wine labels, the aroma fingerprint (Supplementary Fig. S1) could be printed on the package, which would help consumers promptly, accurately and comprehensive understand the aroma characters.

Table grapes have been characterized mainly by herbaceous, fruity, sweet, floral, balsamic and fatty series, the latter being abundant only in some cultivars. However, it remains unclear which components substantially contribute to the aroma evaluation, both positively and negatively. Unlike the good progress of volatiles communication between plant and herbivore^46^,^47^, the relationship between aroma chemical components and people perception has always been a difficult and inadequately addressed problem in this field. Although many studies^48^,^49^ have addressed this problem, it remains unclear and is the bottleneck blocking aroma research. In the present work, on the one hand, sensory evaluation results (Supplementary Fig. S5) showed Shine Muscat (E) was the favourite cultivar. On the other hand, based on the classification results (Figs 5 and 6), we found that Shine Muscat (E) was discriminated from the other groups due to its high OAVs of fatty and balsamic series. In addition, Shine Muscat (E) were mainly represented milk and butter flavours by both assessors of sensory evaluation and market feedback results responses, and these aroma descriptions were also consistent with the fatty and balsamic series. This finding indicates that the milk and butter aromas, which were contributed by the fatty and balsamic series, explain the preference for Shine Muscat and that the fatty and balsamic series are the preferred aromatic series of table grapes. To our knowledge, this is the first report on preferred aromatic series and their components: β-ionone and octanal (Table 5) are important indicators for evaluating or determining the aromatic flavour quality.

Odours that can be described as green, grass, leaves notes, or herbaceous, which were chiefly contributed by C_6_ compounds, typically have negative effects on wine grapes and final wines^5^,^11^. To minimize the herbaceous character, C_6_ compounds possess a higher propensity to form fruity esters during berry development and fermentation process of wine^11^,^17^,^20^. In this study, Shine Muscat and other samples showed high values of herbaceous series (Fig. 3), but these berries did not have any undesirable flavour, which conflicts with previous reports^11^. The main reasons may be summarized as follows: 1, Compounds that are related to herbaceous aroma can also contribute to flesh flavour^50^ (Table 5) in addition to the green, grass and leaves aroma. 2, The depressive or synergic effects of multiple odours^9^,^12^, resulting from the interactions of the various molecules present in grapes, may change the herbaceous aroma into another, more desirable aroma. 3, Herbaceous aroma may help to reduce the greasy feeling produced by strong fatty and balsamic flavours. In addition, to our knowledge, there are differences in taste preferences between Chinese and Western consumers.

Currently, the cultivated area and total yield of wine grapes are much larger than those of table grapes, and berries used to brew wine are subjected to crushing, fermentation and ageing processes. Many studies on the aromas of wines and wine grapes have been conducted, showing that grape berries are rich in floral and herbaceous aromas and somewhat rich in spicy aroma and that these aromas are stronger in skin than in pulp^4^,^12^,^17^. Bound-form volatiles, which are odourless, can be transformed into volatile compounds by hydrolysis during winemaking. Thus, the winemaking process enhances and changes the flavours. Finally, wines are characterized mainly by high intensities of fruity, floral, sweet, fatty, spicy and roasty aromas^9^,^16^,^17^,^39^. However, in this study, table grapes were characterized primarily by herbaceous, fruity, floral, sweet, and balsamic aromatic series (Fig. 3). Compared with wine grapes, which are rich in floral, herbaceous and spicy aromas, table grapes show higher intensities of fruity, sweet and balsamic aromas. This characteristic of table grapes is mainly due to their high contents of β-damascenone, β-ionone and abundant esters, which contributed to the floral, baked apple, violet, balsamic and other flavours (Table 5). In addition, wine grapes generally contain specific aroma compounds, such as monoterpenols in Muscat varieties, methoxypyrazines in Cabernet, C_13_-norisoprenoids in Chardonnay, volatile thiols in Sauvignon, volatile phenols in Traminer, and dimethyl sulfides in Syrah^21^. After the brewing processes, wines also possess a spicy, roasty aroma, which is contributed by eugenol, vanillin, 4-ethylguaiacol and guaiacol^29^, as well as an earthy note, which is contributed by 1-octen-3-ol^51^ and lactone^52^. However, those compounds were absent or present only at very low levels in table grapes, with only monoterpenols and C_13_-norisoprenoids detectable in table grapes.

In conclusion, our study demonstrated that C_6_ volatile compounds were the basic background volatiles in table grape berries, and the total contents of volatiles of pulp and skin largely depended on the levels of esters and terpenes, respectively. C_6_ compounds, terpenes and C_13_ compounds were the most important contributors to berry aroma. For the aroma series, table grapes were characterized mainly by herbaceous, fruity, sweet, floral and balsamic series. Fatty and balsamic series were the preferred series, and the contents of β-ionone and octanal may be useful as key indicators for the improvement of breeding and cultivation measures for table grapes. Finally, table grapes had higher floral, sweet and balsamic flavours than wine grapes but showed no or very weak spicy, roasty and earthy flavours, which are rich in wines.

## Methods

### Plant material and sample collection

A total of 20 table grape cultivars, including the most popular and predominant table grape cultivars in China (Fig. 1), were collected from a single vineyard (Shanghai, China) from July to September 2015. Among them, nine cultivars were *V. vinifera*, including Italian (C), Seto Giants (D), Red Alexandria (F), Rizamat (G), Heibaladuo (I), Black Swan (N), High Bailey (R), Tamina (S), and Centennial Seedless (T), and eleven cultivars were hybrids between *V. vinifera* and *V. labrusca*, including Jumeigui (A), Fujiminori (B), Shine Muscat (E), Zuijinxiang (H), Yoho (J), Oriental Star (K), Kyoho (L), Suiho (M), Jingya (O), Black Beet (P) and Gold Finger (Q). Five-year-old grapevines were cultivated under the same management conditions, including training, pruning, support form, irrigation, soil and fertility. Although many factors, such as climate, temperature and varieties, can influence the quality of grape berries, we can assume that in this study, samples were affected only by cultivar. For each cultivar, ten healthy clusters (approximately five kilograms) from five vines (taking into account the number of berries per cluster and the balance between shadow and sun exposure) were randomly picked at the appropriate commercial harvest date. After sampling, the clusters were taken to the laboratory at 4 °C. Three clusters were subjected to standard chemical analysis; four clusters were used for sensory analysis; and the other clusters were immediately stored at −80 °C until the aroma analysis.

### Chemicals and reagents

Methanol (HPLC grade) was purchased from Merck (Darmstadt, Germany) NaCl (analytical grade) was purchased from Sinopharm Chemical Reagent Co., Ltd (Beijing, China); and pure water was obtained from the Milli-Q purification system (Millipore, Bedford, MA).

The chemical standards were purchased as follows: (***Z***)-3-hexenal, 2-octanol, hexanal, (***E***)-2-hexenal, geranic acid, phellandrene, β-myrcene, β-damascenone, D-limonene, citronellol, benzyl alcohol, neral, α-terpineol, geranial, geraniol, rose oxide II (cis), rose oxide I (trans), phenylethyl alcohol from Sigma (St. Louis, MO, USA); octanoic acid, pentanal, octanal, nonanal, benzaldehyde, 3-methylbutanal, hexanoic acid, (***Z***)-3-hexenol, 1-octen-3-ol, heptanol, ethyl acetate, ethyl butyrate, ethyl isobutyrate, butyl acetate, ethyl pentanoate, methyl salicylate, hexyl acetate, (***E***)-2-hexenoic acid, P-cymene, terpinolene, linalool, 4-terpineol, geranyl acetone from Dr. Ehrenstorfer (Germany); β-ionone from Fluka (Buchs, Switzerland); ethyl hexanoate from Nu-chek (USA); octanol, hexanol, (***E***)-2-hexenol from Chem Service (USA), and n-alkanes (C_7_-C_27_) from Supelco (Bellefonte, PA).

The automatic solid-phase microextraction (SPME) fibres of 50/30 μm divinylbenzene/carboxen/polydimethylsiloxane (DVB/CAR/PDMS) were purchased from Supelco (Bellefonte, PA, USA).

### Sample preparation

Thirty-six berries from different positions (top, middle and bottom) of three clusters (twelve berries per cluster) were randomly collected as a replicate. The thirty-six berries from each sample replicate were thoroughly rinsed with distilled water and dried with filter paper. Then, the berries were squeezed, centrifuged at 10,000 *g* for 10 min at 4 °C (ALC 4239R) and filtered on cellulose paper (Waterman, #1). The juice was analysed for total soluble solids (TSS) using a refractometer (Master-M, Atago, Tokyo, Japan) and for total acidity (TA) by titration with NaOH followed by pH analysis. These procedures were performed in triplicate for each sample.

Prior to the analysis of volatiles, frozen berries of different cluster positions (top, middle and bottom) were divided into three batches of 200 g per cultivar. After thawing overnight at 4 °C, the berries of each batch were manually and carefully peeled, destemmed and deseeded. Skins were centrifuged (4000 rpm, 5 °C, 20 min) to separate them from must, and they were then dried with filter paper and weighed to calculate the skin/pulp ratio. Subsequently, to decrease oxidation, the skins were immediately immersed into bottles containing extraction solutions (pH 3.2, 3 g/L of tartaric acid, 50 mg/L of Vc), which were prepared as previously described by Alessandro Genovese^12^ with slight modifications. After extraction and centrifugation, the extracted solutions were used for volatile analysis.

Deseeded pulps were homogenized (Black & Decker, CJ60) for 1 min after the addition of 50 mg/L Vc, centrifuged at 10,000 *g* for 10 min at 4 °C (ALC 4239R), and filtered on cellulose paper, and the pulp juice was obtained for volatile analysis. Pulp juice was adjusted to pH 3.9 to obtain the same matrix as soon as possible, and the volume of juice was measured to calculate the concentrations of aromatic compounds expressed in μg/kg.

### HS-SPME procedure

For headspace solid phase microextraction (HS-SPME), the extraction of aroma volatiles was performed using a Gerstel MPS-2 autosampler (Gerstel). Prior to use, the SPME fibre was conditioned according to the manufacturer’s recommendations. Then, the fibre was conditioned for 10 min per day at 250 °C. For the HS-SPME assay, after the deep optimization (Supplementary Fig. S6), including selection of the fibre coating, extraction time, extraction temperature and the sample volume/headspace volume ratio, 6 mL of sample (pulp juice or skin extract solution) was transferred to a 20 mL glass vial. After the additional of sodium chloride (1.5 g) and 2-octanol internal standard solution (5 μL, 155 mg/L), corresponding to a ratio of the volume of the liquid phase to the headspace volume (1/β) of 0.5^53^, the vial was capped with a PTFE septum and an aluminium cap (Chromacol, Hertfordshire, UK). The Gerstel MPS2 autosampler was operated in the SPME mode with a DVB/CAR/PDMS (50/30 μm) fibre. Volatile compounds were equilibrated by agitating the sample (250 rpm) for 10 min at 50 °C and then extracted for 30 min at the same temperature and agitation. After the extraction, the fibre was immediately inserted into the gas chromatography (GC) injection port to desorb volatiles at 260 °C for 3 min in splitless mode. All measurements were performed in triplicate.

### GC-MS analysis

The desorbed volatile compounds were separated in an Agilent 7890 GC (Agilent Technologies, Santa Clara, CA, USA) equipped with HP-INNOWAX (30 m × 0.25 mm i.d., 0.25 μm film thickness; J & W scientific, USA) and coupled with an Agilent 5975 mass spectrometer. The carrier gas was helium, provided at a flow rate of 1 mL min^−1^. The GC oven temperature was programmed as follows: 40 °C for 5 min, increased to 240 °C at 5 °C min^−1^, and then ramped at 20 °C min^−1^ to 260 °C and held there for 5 min.

For the mass spectrometry (MS) system, the temperatures of the transfer line, the quadrupole and the ionization source were 260, 150 and 230 °C, respectively; electron impact mass spectra were recorded at 70 eV ionization voltages. The acquisitions were performed in full-scan mode (20–400 m/z).

Identification. Retention indices (Table 5) were calculated after analysing C_7_-C_27_ n-alkane under the same chromatographic conditions. Compound identifications were based on matching mass spectra from the standard NIST 2011 library and on retention indices of the authenticated standards. When the authenticated standards were not available, tentative identifications were based on the standard national institute of standards and technology (NIST) 2011 library and a comparison of retention indices reported in the literature.

Quantification. The quantification was modified from methods reported in previous studies. Based on the average concentrations of sugars^54^ and acids and the pH in pulp juice (Fig. 1), calibration graphs were built in blank solutions for skin (pH 3.2, 3 g/L of tartaric acid, 50 mg/L of Vc) and juice (pH 3.9, 100 g/L fructose, 100 g/L glucose, 3 g/L tartaric acid, 50 mg/L of Vc) spiked with the authenticated standards and the internal standard. The linearity of calibration curves (r^2^) was satisfactory in almost all cases (Supplementary Table S5). It is worth mentioning that quantitative effectiveness of SPME vary from matrix to matrix in the solution, and is particularly vulnerable and suspicious sometimes. After all, this system is not often quantitative and not all of the compounds determined by SPME can realize the satisfactory quantification in any matrix solution especially when multiple chemicals are trapped together. Therefore, SPME, especially for quantification, must be fully verified before making a decision according to the actual situation (matrix). In this study, a valid method, in which various known concentrations of standards were spiked into the pulp juice or skin extraction solutions on different days, was selected for verifying the validity of this method. For each target compound, the three added concentrations corresponded to 100%, 50%, and 10% of the near maximum concentration found in 20 tested table grapes in this work. The validation parameters were recovery and reproducibility. Recovery (%) = [concentration determined in spiked solution]/[concentration determined in solution + added concentration] × 100. The reproducibility of the method was estimated by the relative standard deviation (r.s.d.) of four analyses on four different days for each compound. Each spiked solution was extracted using the SPME procedure in duplicate, for a total of four gas chromatography mass spectrometry (GC-MS) analyses for each sample of skin and juice. Most of the r.s.d. (%) values were below 10% and showed good reproducibility in both skin and pulp juice (Supplementary Table S6). Except for acids (Supplementary Table S6), the recoveries of most compounds were appropriate (70–120%). Therefore, in this work, except for acids, the quantitative effectiveness of the aroma compounds is satisfactory. As for acids, although their recovery rates are low, their concentrations are low in table grapes. More importantly, their odour thresholds are too high (≥1000 ppb), making their OAVs was much lower than 1. Therefore, the difficulty in quantifying acids has little impact on the results of this study, especially for OAVs. In conclusion, through the SPME optimization and maximum matching matrix, the quantitative effectiveness error of this method is within a controllable range and is satisfactory. For the compounds without calibration curves, semi-quantitative determinations were obtained using the internal standard.

### Calculation of the aromatic series values

Descriptors and thresholds for the aroma compounds can be obtained (Table 5) from previous reports. Based on its aroma descriptors, one compound could be included in one or several aromatic series. In the present study, all volatile compounds were classified into ten aromatic series: herbaceous, floral, fruity, sweet, spicy, roasty, fatty, earthy, balsamic and solvent (Table 5).

### Sensory analysis

Because the samples used in this work were the most popular and predominant table grape cultivars in China, their general aroma evaluation and popularity with the public were obtained from market feedback. To further obtain accurate, professional information, sensory evaluation analyses were performed in a standard sensory-analysis chamber (ISO 8589, 1998) equipped with separate booths. There was a uniform source of lighting and absence of noise and distracting stimuli, and the ambient temperature was between 23 and 25 °C throughout the day. After a general training (we explicitly told the assessors to record the aroma preference intensity of samples), the samples were evaluated by a group of 36 assessors (16 females and 30 males) ranging in age from 19 to 55 years. After sampling, rinsing and drying, grape berries were kept for 1 hour in the sensory-analysis chamber to reach the ambient temperature (23–25 °C) before testing. After smelling the grape berries, the assessors took a bite of each sample (approximately 60 g for one assessor), chewed it well and swallowed it. The assessors were free to take as many bites as necessary to complete the assessments, and they then rated and noted the description and specific aroma preference intensity of each cultivar on a five-point scale, where values 1–5 indicated the aroma consumer preference intensity as very low, low, medium, high and very high, respectively. After finishing each sample, assessors were instructed to take a bite of an unsalted cracker and a sip of water between samples. The results are shown in (Supplementary Fig. S5) and are almost the same as the results based on market feedback.

### Statistical analysis

One-way analysis of variance (ANOVA) and hierarchical cluster analysis (HCA) were performed using SPSS 19.0; principal component analysis (PCA) was performed using SIMCA-P 11.5. PCA and HCA were weighted by using unit variance (UV) and zero-mean normalization scaling, respectively. The other figures were made using Origin 9.1. All the data in this experiment are presented as the average of three replicates.

## Additional Information

**How to cite this article**: Wu, Y. *et al*. Aroma characterization based on aromatic series analysis in table grapes. *Sci. Rep.* **6**, 31116; doi: 10.1038/srep31116 (2016).

## Supplementary Material

Supplementary Information

## Figures and Tables

**Table 1:**
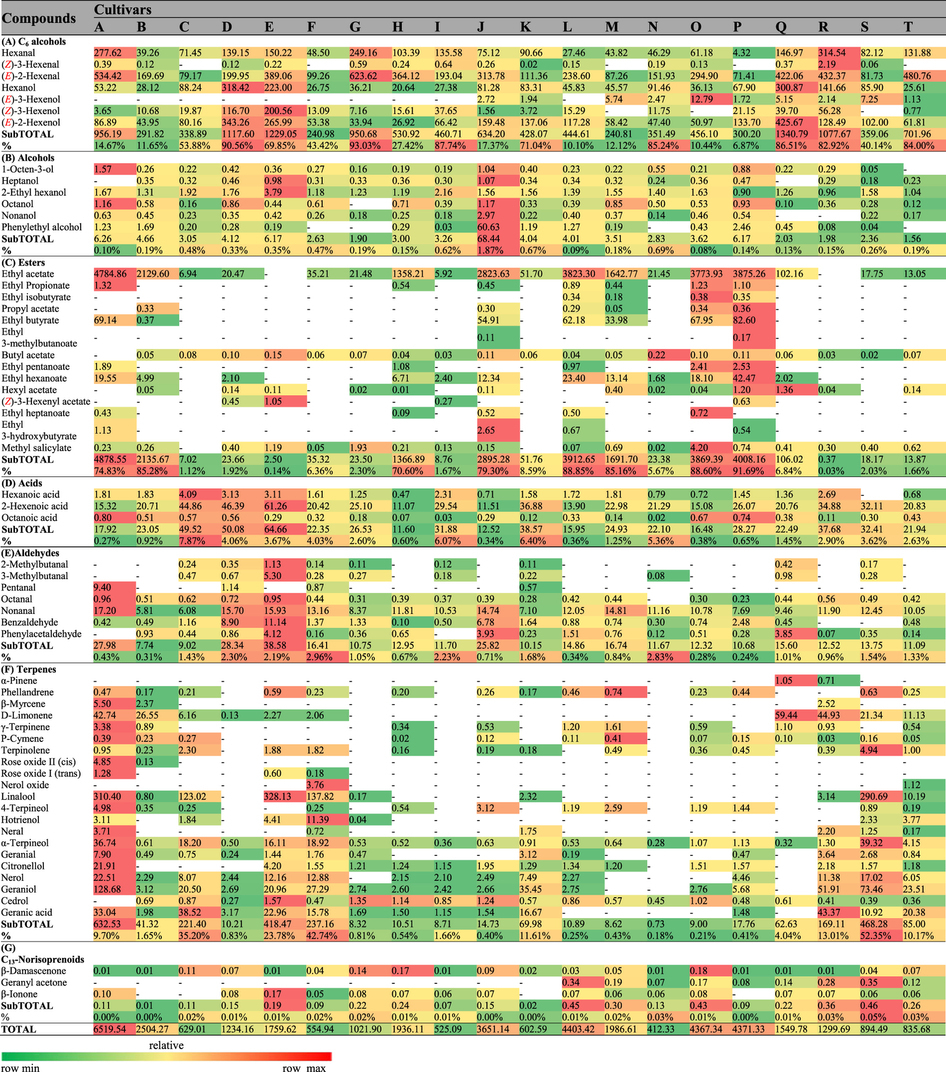
Concentrations (μg/kg) of volatile compounds determined in the pulp juice of table grapes.

Data are means (n = 3). The capital letters refer to the table grape cultivars listed in Fig. 1. The aroma compounds were listed on the left of the concentration arrays, and the colour scale was shown at the bottom. The higher concentration for each compound was presented in red; otherwise, green was used; - Indicated that the compound was not detected.

**Table 2:**
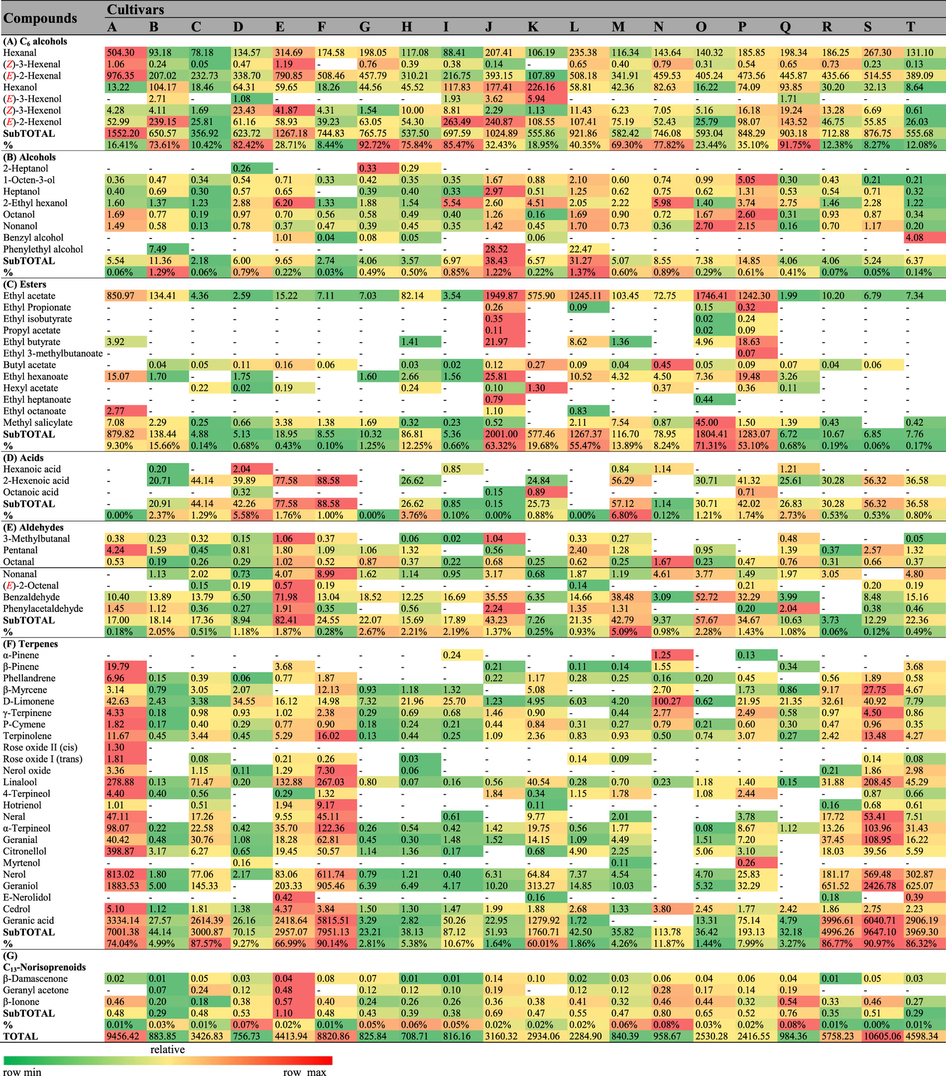
Concentrations (μg/kg) of volatile compounds determined in the skin of table grapes.

Data are means (n = 3). The capital letters refer to the table grape cultivars listed in Fig. 1. The aroma compounds were listed on the left of the concentration arrays, and the colour scale was shown at the bottom. The higher concentration for each compound was presented in red; otherwise, green was used; -Indicated that the compound was not detected.

**Table 3:**
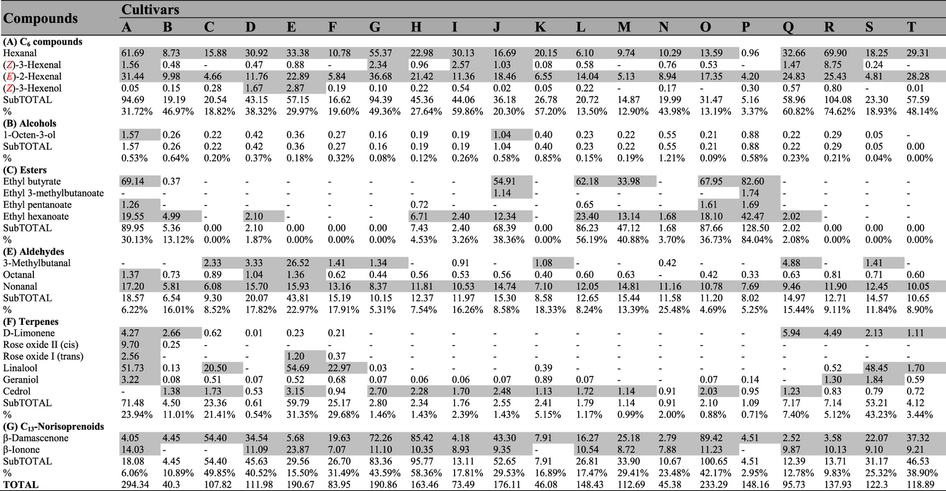
Odor activity values (OAVs) of active volatile compounds determined in the pulp juice of table grapes.

Data are means (n = 3). -Indicated that the compound was not detected. Gray represents the value equal to or greater than 1. The capital letters refer to the table grape cultivars listed in Fig. 1.

**Table 4:**
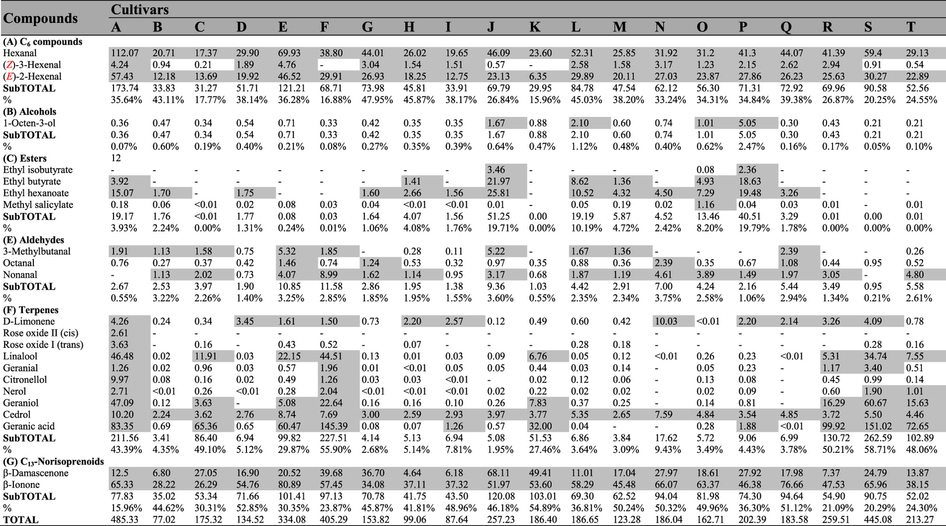
Odor activity values (OAVs) of active volatile compounds determined in the skin of table grapes.

Data are means (n = 3). -Indicated that the compound was not detected. Gray represents the value equal to or greater than 1. The capital letters refer to the table grape cultivars listed in Fig. 1.

**Table 5:** Chemical standards, linear retention indices (LRI), odour descriptors, odorant series, odour thresholds (ppb in water) of the studied compounds.

Compounds	LRI	Odour threshold (μg/l)	Odour descriptor	Odorant series
C_6_ compounds				
Hexanal	1105	4.5^55^	Green^38^	1
(***Z***)-3-Hexenal	1148	0.25^51^	Grass^42^	1
(***E***)-2-Hexenal	1226	17^12^	Grass^12^, herbaceous^12^	1
Hexanol	1355	500^55^,^56^	Flower^12^,^16^,^39^, green^12^,^16^,^39^, cut grass^16^,^39^, grass^12^, herbaceous^12^,^30^,^40^, wood^12^,^30^,^40^	1,2
(***E***)-3-Hexenol	1365	1000^57^	Green^29^,^30^, bitter^29^, fatty^29^, herbaceous^30^, fresh^57^	1,7
(***Z***)-3-Hexenol	1385	70^55^	Grass^12^,^42^,^58^, herbaceous^12^,^30^, green^12^,^14^,^16^,^39^,^41^, fatty^12^,^14^,^29^, bitter^12^,^14^,^29^	1,7
(***E***)-2-Hexenol	1408	100^50^	Herbaceous^12^,^30^, green^12^,^30^,^50^	1
Alcohols				
2-Heptanol	1324	70^50^	Fruity^50^, herbaceous^50^	1,3
1-Octen-3-ol	1453	1^45^	Mushroom^38^,^45^	8
Heptanol	1456	425^55^	Oily^16^	7
2-Ethyl hexanol	1490	270^59^	Floral^60^	2
Octanol	1557	110^61^	Jasmine^40^, lemon^40^	2
Nonanol	1659	50^61^	Rose-orange^50^	2
Benzyl alcohol	1873	10000^10^	Roasted^12^, toasted^12^, sweet^12^,^16^, fruity^12^,^16^	3,4,6
Phenylethyl alcohol	1908	1100^10^	Floral^12^,^16^, rose^12^,^16^, honey^12^	2
Esters				
Ethyl acetate	889	5000^55^	Pineapple^30^,^62^, fruity^16^,^30^, solvent^16^,^30^, anise^62^, balsamic^40^	3,5,7,9,10
Ethyl propionate	953	10^55^	Banana^40^, apple^40^	3
Ethyl isobutyrate	962	0.1^55^	Fruity^40^	3
Propyl acetate	973	4700^14^	Celery^14^	1
Ethyl butyrate	1042	1^55^	Fruity^16^	3
Ethyl 3-methylbutanoate	1077	0.1^55^	Fruity^38^	3
Butyl acetate	1082	66^55^	Fruity^14^	3
Ethyl pentanoate	1143	1.5^51^	Grass^42^	1
Ethyl hexanoate	1241	1^55^	Fruity^14^,^40^, green apple^14^,^16^,^39^,^40^, banana^14^,^40^, wine-like^14^,^40^, brandy^14^	3
Hexyl acetate	1278	670^14^,^30^	Apple^14^,^30^, pear^14^,^30^,^58^, floral^14^,^16^,^30^,^39^, green^16^,^39^, cherry^14^,^58^	1,2,3
(***Z***)-3-Hexenyl acetate	1321	750^63^	Fruity^63^, green leaves^63^	1,3
Ethyl heptanoate	1337	2^55^	Winelike^29^, brandy^29^, fruity^29^	3,10
Ethyl octanoate	1438	194^55^	Sweet^16^,^40^, floral^40^, fruity^16^,^40^, banana^40^, pear^40^, brandy^40^	2,3,4
Ethyl 3-hydroxybutyrate	1518	20000^58^	Grape^29^, fruity^41^, caramel^29^, toasted^29^	3,4,6
Acids				
Hexanoic acid	1846	3000^64^	Rancid^40^, cheese^40^, fatty^40^, sweat^16^	7
2-Hexenoic acid	1969	1000^50^	Fatty^50^, rancid^43^,^50^	7
Octanoic acid	2061	3000^55^	Rancid^40^, cheese^16^,^40^, fatty^40^, sweat^16^	7
Aldehydes				
2-Methylbutanal	912	1.3^50^	Green^50^, malty^50^	1
3-Methylbutanal	915	0.2^64^	Fresh grass^50^, cocoa^50^	1
Pentanal	976	12^55^,^61^	Fat^65^, green^65^	1,7
Octanal	1292	0.7^51^,^55^,^61^	Honey^29^, green^29^,^43^, fatty^29^, fruity^38^, citrus^38^, lemon^42^,^43^, fat^43^, soap^43^	1,2,3,7
Nonanal	1395	1^55^,^61^	Fat^43^, citrus^43^, green^43^, fruity^38^	1,3
(***E***)-2-Octenal	1430	3^55^,^56^,^61^	Green^43^, nut^43^, fat^43^	1
Benzaldehyde	1523	350^55^	Sweet^16^,^39^, fruity^16^,^39^, roasted^30^, almond^14^,^30^,^58^, fragant^14^, burnt sugar^58^	2,3,4,6
Phenylacetaldehyde	1643	4^55^	Flowery^42^, rose^42^	2
Terpenes				
α-Pinene	1018	6^55^	Pine^50^, resinous^50^	1
β-Pinene	1101	140^55^	Woody^50^, resinous^50^	1
Phellandrene	1164	40^55^	Sweet^50^, rose-like^50^	2,4
β-Myrcene	1169	36^10^	Green burning^38^, green^38^	1,6
D-Limonene	1197	10^10^	Fruity^44^, lemon^44^	3
γ-Terpinene	1246	1000^55^	Fruity^50^, lemon-like^50^	3
P-Cymene	1271	11.4^55^	Citrus^38^, green^38^	1,3
Terpinolene	1282	200^55^	Piney^66^	1
Rose oxide II (cis)	1353	0.5^10^	Floral^38^, lychee-like^38^, rose^37^	2
Rose oxide I (trans)	1367	0.5^10^	Rose^37^	2
Nerol oxide	1471	3000^67^	Oil^67^, flower^67^	2,7
Linalool	1548	6^10^	Citrus^14^,^40^,^41^, floral^14^,^16^,^40^,^41^, sweet^14^,^40^,^41^, grape-like^14^,^40^,^41^	2,3,4
4-Terpineol	1601	130^55^	Flowers^44^,^57^, nutmeg^57^, moldy^30^	1,2,5
Hotrienol	1611	110^68^	Fresh^68^, floral^68^, fruity^68^	1,2,3
Neral	1681	1000^55^	Fruity^40^	3
α-Terpineol	1696	330^10^	Lilac^14^,^41^, floral^14^,^41^, sweet^14^,^41^	2,4
Geranial	1732	32^55^	Citrus^38^, citric fruit^38^	3
Citronellol	1765	40^10^	Rose^40^,^58^	2
Myrtenol	1789	7^50^	Flowery^50^, mint^50^	1,2
Nerol	1800	300^10^	Flower^58^, grass^58^, floral^29^, green^29^	1,2
Geraniol	1853	40^10^	Citric^12^, floral^12^,^41^, orange flower^12^,^41^, roses^12^,^16^,^39^,^58^, geranium^12^,^16^,^39^,^58^	2
E-Nerolidol	2040	250^55^	Rose^14^,^29^, apple^14^,^29^, green^14^,^29^, citrus^14^, waxy^29^, woody^29^	1,2,3,10
Cedrol	2126	0.5^45^	Cool^45^, camphor^43^	1
Geranic acid	2337	40^17^	Green^17^	1
C_13_-Norisoprenoids				
β-Damascenone	1821	0.002^64^	Sweet^16^,^38^,^39^, fruity^16^,^39^, floral^38^, honey^38^, baked apple^42^	2,3,4
Geranyl acetone	1854	60^69^	Fresh^69^, floral^69^	1,2
β-Ionone	1935	0.007^55^,^64^	Balsamic^14^, rose^14^, violet^14^	2,9

LRI, linear retention indices on a HP-INNOWAX column. The odour thresholds and odour descriptors were reported in literature. Compounds determined in water solution, except for 2-ethyl hexanol, propyl acetate, hexyl acetate, ethyl 3-hydroxybutyrate determined in ethanol-water solution; (***Z***)-3-hexenyl acetate determined in sunflower oil; geranic acid (not found) was assumed the same as geraniol; hotrienol not found the media. Odorant series:1, herbaceous; 2, floral; 3, fruity; 4, sweet; 5, spicy; 6, roasty; 7, fatty; 8, earthy; 9, balsamic; 10, solvent.
