# Supplementary material for: Aroma characterization based on aromatic series analysis in table grapes
Source: Sci Rep. 2016 Aug 4;6:31116. doi: 10.1038/srep31116 (PMC4973247; doi:10.1038/srep31116)
Supplement: Supplementary Information [file srep31116-s1.pdf]

Aroma characterization based on aromatic series analysis in table grapes

Yusen Wu, Shuyan Duan, Liping Zhao, Zhen Gao, Meng Luo, Shiren Song, Wenping Xu, Caixi Zhang, Chao Ma*, Shiping Wang*

Department of Plant Science, School of Agriculture and Biology, Shanghai Jiao Tong University, Shanghai, China.

*** Corresponding author:** Department of Plant Science, School of Agriculture and Biology, Shanghai Jiao Tong University, Shanghai, China.
Tel& Fax: +0086-21-34205961
E-mail address: fruit@sjtu.edu.cn, chaoma2015@sjtu.edu.cn

Supplementary information
Figures

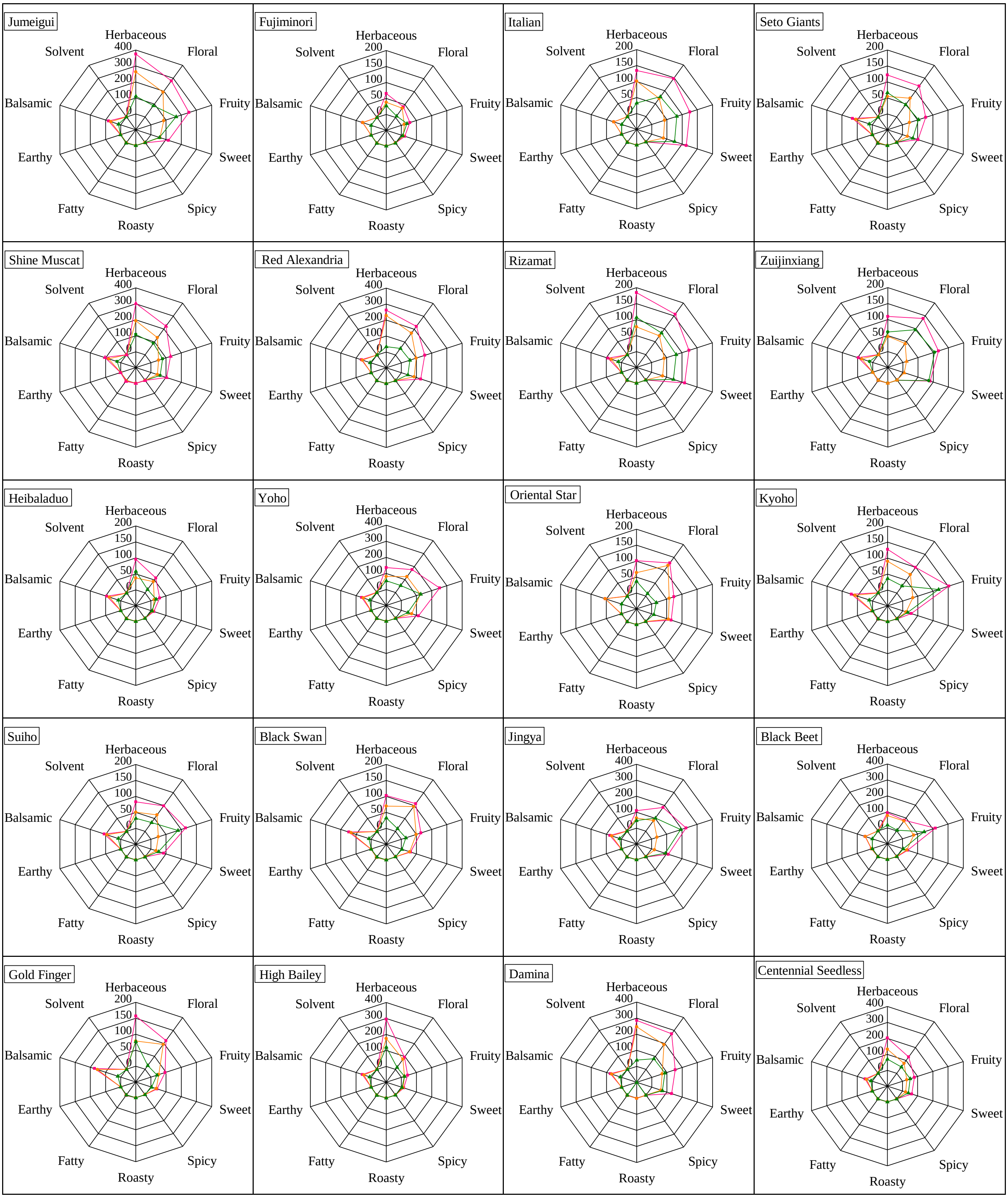

Figure S1. Aromatic fingerprints of table grapes. Green line with triangle indicated the fingerprints of pulp juice; yellow line with dot indicated the fingerprints of skin; wine red line with square indicated the fingerprints of whole berries. Scale range of nine cultivars (Jumeigui, Shine Muscat, Red Alexandria, Yoho, Jingya, Black Beet, High Bailey, Tamina, Centennial Seedless) was 0-400; the others was 0-200.

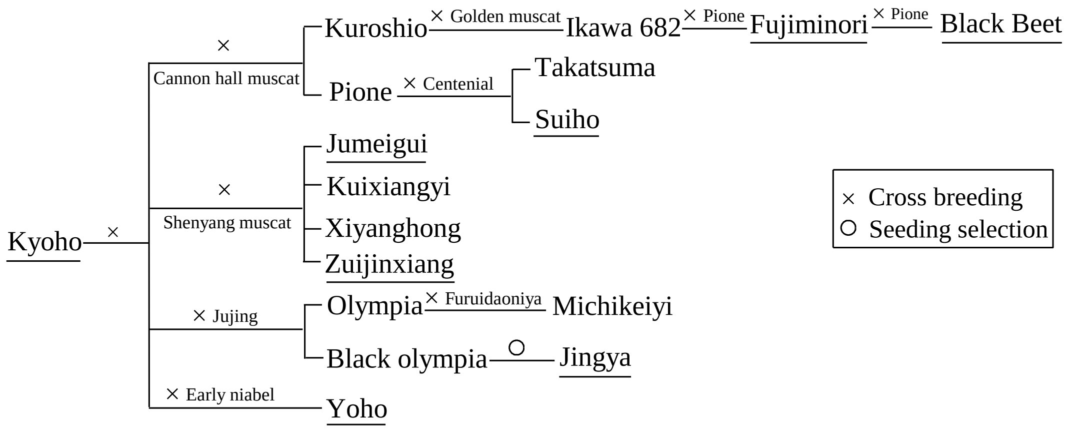

Figure S2. Part pedigree chart of Kyoho grapevine series (Kyoho or its offspring). The eight cultivars (A, B, H, J, L, M, O, P) in this study were underlined. Cross indicated cross breeding; circle indicated seeding selection.

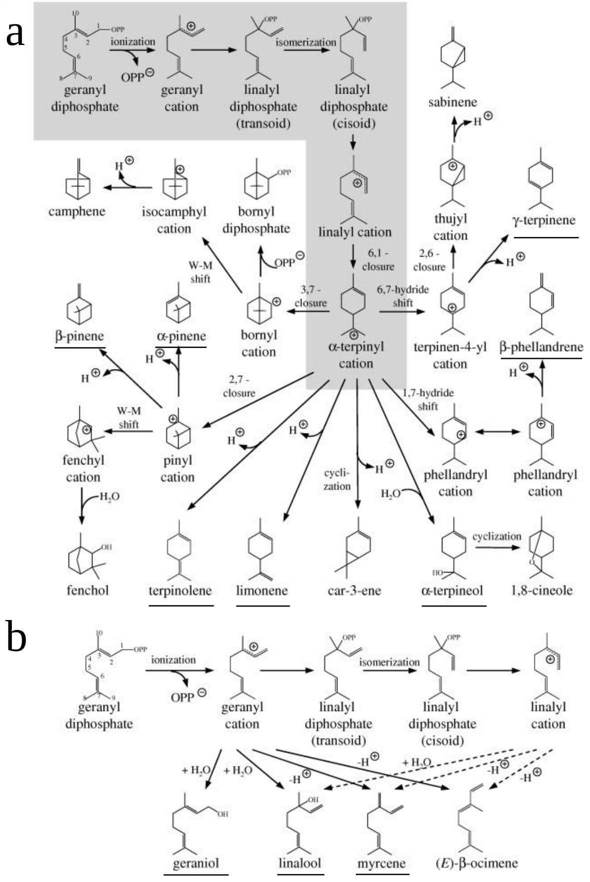

Figure S3. Synthesis mechanisms of monoterpenes in grapes obtained from the previous report[25]. (a) indicated synthetic pathway of cyclic monoterpenes; **(b)** indicated synthetic pathway of acyclic monoterpenes. The mainly compounds in this study are underlined, including cyclics (α-pinene, β-pinene, phellandrene, limonene, γ-terpinene, α-terpineol, terpinolene) and acyclics (linalool, β-myrcene, geraniol).

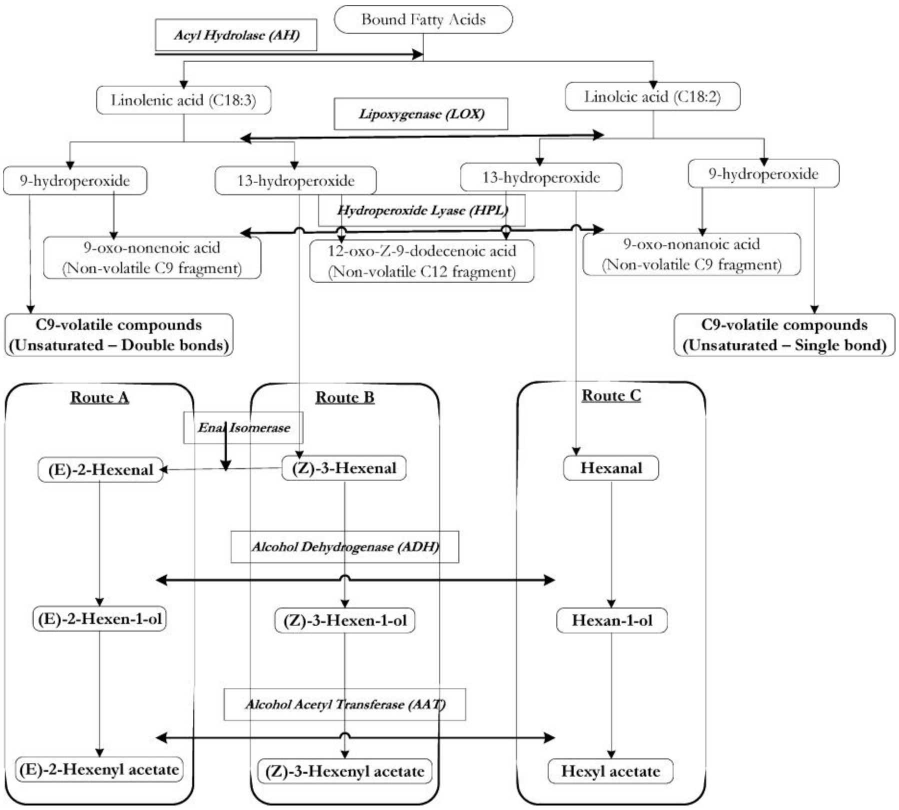

Figure S4. Metabolic pathway of C_6 compounds in grapes obtained from the previous report[11,20]. Route A, B, C indicated the different metabolic pathways, respectively.

	Herbaceous	Floral	Fruity	Sweet	Spicy	Roasty	Fatty	Earthy	Balsamic	Solvent	
A	374.88	279.68	251.36	116.22	1.21	0.22	4.28	1.93	80.18	1.34	4.47
B	65.99	41.84	29.02	11.74	0.33	0.08	1.72	0.73	28.53	0.31	1.97
C	134.46	146.81	125.30	114.04	0.01	0.13	1.70	0.56	26.29	0.00	4.19
D	122.07	120.08	76.79	51.54	0.00	0.10	3.74	0.96	65.86	0.00	4.19
E	302.77	221.39	128.66	103.46	0.00	0.24	6.68	1.07	104.76	0.00	4.81
F	262.38	221.24	154.37	127.38	0.08	0.38	1.88	0.60	64.52	0.01	4.33
G	184.50	156.60	123.20	109.18	0.00	0.08	1.92	0.58	45.18	0.00	3.56
H	109.77	139.46	117.17	90.36	0.30	0.07	1.86	0.54	47.75	0.33	3.92
I	96.15	58.02	29.28	10.38	0.00	0.09	1.54	0.54	46.25	0.00	2.31
J	136.43	177.16	251.58	112.64	1.08	0.12	2.61	2.71	62.28	1.61	4.36
K	101.82	128.66	74.11	64.70	0.13	0.16	1.04	1.28	53.73	0.13	3.94
L	127.50	99.61	151.88	28.39	1.07	0.05	3.15	2.33	69.85	1.27	3.53
M	83.29	98.93	114.65	42.85	0.46	0.11	1.56	0.83	54.54	0.35	3.94
N	102.66	107.45	65.11	30.69	0.02	0.08	2.70	1.29	73.97	0.02	3.92
O	109.81	184.05	226.74	109.55	1.23	0.15	2.04	1.20	75.34	1.68	3.89
P	94.59	81.91	217.08	33.94	1.19	0.15	2.67	5.93	47.40	1.02	3.83
Q	156.87	110.96	47.00	20.44	0.02	0.04	2.75	0.52	86.55	0.02	3.94
R	295.03	94.88	42.09	16.84	0.00	0.32	2.31	0.71	57.65	0.00	3.97
S	285.39	273.57	154.10	130.58	0.02	0.79	2.09	0.26	75.07	0.00	4.33
T	201.98	126.95	78.97	60.61	0.01	0.17	1.34	0.21	47.36	0.00	4.19

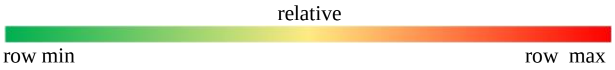

Figure S5. The relationship between aromatic series values (whole berries) and aroma preference intensity scores (scale: 1-5) obtained from sensory evaluation analysis. Data were means (n=3). Ten aromatic series were shown across the top and the colour scale was shown at the bottom. The higher values for each aromatic series were presented in red; otherwise, green was used; white represents the value less than 1. Capital letters (right) referred to the table grape cultivars listed in Figure 1, and their aroma preference scores (left) were shown.

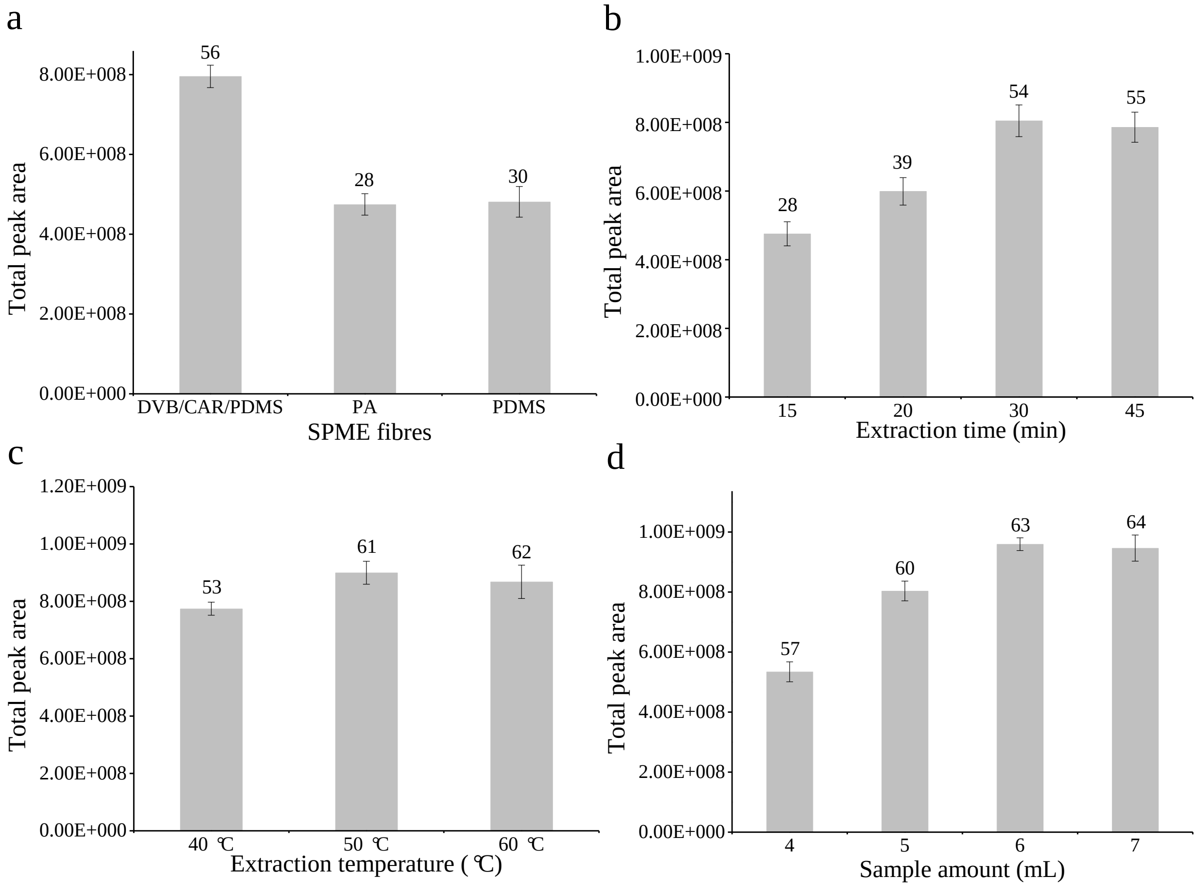

Figure S6. The optimization of headspace solid phase microextraction (HS-SPME) method. Performance characteristics obtained for tested solid phase microextraction (SPME) fibres (a) during 40 min at 40 ℃, extraction time (b) using divinylbenzene/carboxen/polydimethylsiloxane (DVB/CAR/PDMS) fibre at 40 ℃, extraction temperature (c) using DVB/CAR/PDMS fibre during 30 min and volume/headspace volume ratio (d) using DVB/CAR/PDMS fibre during 30 min at 50 ℃ on total aroma peak area in this work. Number indicated the number of kinds of identified compounds. The best results were obtained using 6 mL of sample (pulp juice or skin extraction solutions) in a 20 mL glass vial with addition of 1.5 g of sodium chloride (NaCl) ($1/\beta = 0.5$), and after equilibrating, a DVB/CAR/PDMS (50/30 μm) fibre was selected for extraction at 50 ℃ for 30 min under agitation. Note: in Figure S6 (a), DVB/CAR/PDMS, PA, PDMS indicated divinylbenzene/carboxen/polydimethylsiloxane (50/30 μm), polyacrylate (85 μm), polydimethylsiloxane (100 μm) fiber, respectively.

Tables

Monoterpenes	Cultivars																			
	**A**	**B**	**C**	**D**	**E**	**F**	**G**	**H**	**I**	**J**	**K**	**L**	**M**	**N**	**O**	**P**	**Q**	**R**	**S**	**T**
**Cyclics**																				
α-Pinene	-	-	-	-	-	-	-	-	-	-	-	-	-	-	-	-	1.05	0.71	-	-
Phellandrene	0.47	0.17	0.21	-	0.59	0.23	-	0.20	-	0.26	0.17	0.46	0.74	-	0.23	0.44	-	-	0.63	0.25
4-Terpineol	4.98	0.35	0.25	-	-	0.25	-	0.54	-	3.12	-	1.19	2.59	-	1.19	1.44	-	-	0.89	0.19
D-Limonene	42.74	26.55	6.16	0.13	2.27	2.06	-	-	-	-	-	-	-	-	-	-	59.44	44.93	21.34	11.13
γ-Terpinene	3.38	0.89	-	-	-	-	-	0.34	-	0.53	-	1.20	1.61	-	0.59	-	1.10	0.93	-	0.54
α-Terpineol	36.74	0.61	18.20	0.50	16.11	18.92	0.53	0.52	0.36	0.63	0.91	0.53	0.64	0.28	1.07	1.13	0.32	1.30	39.32	4.15
Terpinolene	0.95	0.23	2.30	-	1.88	1.82	-	0.16	-	0.19	0.18	-	0.49	-	0.36	0.45	-	0.39	4.94	1.00
P-Cymene	0.39	0.23	0.27	-	-	-	-	0.02	-	0.12	-	0.11	0.41	-	0.07	0.15	0.10	0.03	0.16	0.05
SubTOTAL	89.65	29.03	27.39	0.63	20.85	23.28	0.53	1.78	0.36	4.85	1.26	3.49	6.48	0.28	3.51	3.61	62.01	48.29	67.28	17.31
**%**	**14.17%**	**72.20%**	**12.44%**	**6.87%**	**5.02%**	**9.85%**	**7.74%**	**19.20%**	**5.01%**	**35.95%**	**1.82%**	**34.76%**	**84.38%**	**100.00%**	**45.12%**	**20.90%**	**100.00%**	**28.64%**	**14.40%**	**20.48%**
**Acyclics**																				
Roseoxide II (cis)	4.85	0.13	-	-	-	-	-	-	-	-	-	-	-	-	-	-	-	-	-	-
Roseoxide I (trans)	1.28	-	-	-	0.60	0.18	-	-	-	-	-	-	-	-	-	-	-	-	-	-
Nerol oxide	-	-	-	-	-	3.76	-	-	-	-	-	-	-	-	-	-	-	-	-	1.12
Linalool	310.40	0.80	123.02	-	328.13	137.82	0.17	-	-	-	2.32	-	-	-	-	-	-	3.14	290.69	10.19
Hotrienol	3.11	-	1.84	-	4.41	11.39	0.04	-	-	-	-	-	-	-	-	-	-	-	2.33	3.77
Neral	3.71	-	-	-	-	0.72	-	-	-	-	1.75	-	-	-	-	-	-	2.20	1.25	0.17
β-Myrcene	5.50	2.37	-	-	-	-	-	-	-	-	-	-	-	-	-	-	-	2.52	-	-
Geranial	7.90	0.49	0.75	0.24	1.44	1.76	0.47	-	-	-	3.12	0.19	-	-	-	0.47	-	3.64	2.68	0.84
Citronellol	21.91	-	-	-	4.20	1.55	1.21	1.24	1.15	1.95	1.29	1.34	1.20	-	1.51	1.57	-	2.18	1.57	1.18
Nerol	22.51	2.29	8.07	2.44	12.16	12.88	-	2.15	2.10	2.49	7.49	2.27	-	-	-	4.46	-	11.38	17.02	6.05
Geraniol	128.68	3.12	20.50	2.69	20.96	27.29	2.74	2.60	2.42	2.66	35.45	2.75	-	-	2.76	5.68	-	51.91	73.46	23.51
Geranic acid	33.04	1.98	38.52	3.17	22.96	15.78	1.69	1.50	1.15	1.54	16.67	-	-	-	-	1.48	-	43.37	10.92	20.38
SubTOTAL	542.89	11.18	192.70	8.54	394.86	213.13	6.32	7.49	6.82	8.64	68.09	6.55	1.20	0.00	4.27	13.66	0.00	120.34	399.92	67.21
**%**	**85.83%**	**27.80%**	**87.56%**	**93.13%**	**94.98%**	**90.15%**	**92.26%**	**80.80%**	**94.99%**	**64.05%**	**98.18%**	**65.24%**	**15.63%**	**0.00%**	**54.88%**	**79.10%**	**0.00%**	**71.36%**	**85.60%**	**79.52%**
TOTAL terpenes	632.54	40.21	220.09	9.17	415.71	236.41	6.85	9.27	7.18	13.49	69.35	10.04	7.68	0.28	7.78	17.27	62.01	168.63	467.2	84.52
**%[a]**	**100.00%**	**97.31%**	**99.41%**	**89.81%**	**99.34%**	**99.68%**	**82.33%**	**88.20%**	**82.43%**	**91.58%**	**99.10%**	**92.19%**	**89.10%**	**38.36%**	**86.44%**	**97.24%**	**99.01%**	**99.72%**	**99.77%**	**99.44%**

Table S1. Concentrations (μg/kg) of monoterpenes determined in pulp juice of table grapes. Data are shown as the means (n=3). **%[a]** indicated that the percentage of monoterpenes contents account for the total terpenes contents. - indicated that the compound was not detected. Capital letters refer to the table grape cultivars listed in Figure 1.

Monoterpenes	Cultivars																			
	**A**	**B**	**C**	**D**	**E**	**F**	**G**	**H**	**I**	**J**	**K**	**L**	**M**	**N**	**O**	**P**	**Q**	**R**	**S**	**T**
**Cyclics**																				
α-Pinene	-	-	-	-	-	-	-	-	0.24	-	-	-	-	1.25	-	0.13	-	-	-	-
β-Pinene	19.79	-	-	-	3.68	-	-	-	-	0.21	-	0.11	0.14	1.55	-	-	0.34	-	-	3.68
Phellandrene	6.96	0.15	0.39	0.06	0.77	1.87	-	-	-	0.22	1.17	0.28	0.25	0.16	0.20	0.45	-	0.56	1.89	0.58
D-Limonene	42.63	2.43	3.38	34.55	16.12	14.98	7.32	21.96	25.70	1.23	4.95	6.03	4.20	100.27	0.62	21.95	21.35	32.61	40.92	7.79
γ-Terpinene	4.33	0.18	0.98	0.93	1.02	2.38	0.29	0.69	0.68	1.46	0.90	-	0.44	2.77	-	2.49	0.58	0.97	4.50	0.86
P-Cymene	1.82	0.17	0.40	0.29	0.77	0.90	0.18	0.24	0.21	0.44	0.84	0.31	0.27	0.79	0.21	0.60	0.30	0.47	0.96	0.35
Terpinolene	11.67	0.45	3.44	0.45	5.29	16.02	0.13	0.44	0.25	1.09	2.36	0.83	0.93	0.50	0.74	3.07	0.27	2.42	13.48	4.27
4-Terpineol	4.40	0.40	0.56	-	0.29	1.32	-	-	-	1.84	0.34	1.15	1.78	-	1.08	2.44	-	-	0.87	0.66
α-Terpineol	98.07	0.22	22.58	0.42	35.70	122.36	0.26	0.54	0.42	1.42	19.75	0.56	1.77	-	0.08	8.67	1.12	13.26	103.96	31.43
Myrtenol	-	-	-	0.16	-	-	-	-	-	-	-	-	0.11	-	-	0.26	-	-	-	-
SubTOTAL	189.67	4.00	31.73	36.86	63.64	159.83	8.18	23.87	27.50	7.91	30.31	9.27	9.89	107.29	2.93	40.06	23.96	50.29	166.58	49.62
**%**	**2.71%**	**9.32%**	**1.06%**	**53.19%**	**2.16%**	**2.01%**	**37.23%**	**63.84%**	**31.95%**	**16.00%**	**1.72%**	**23.40%**	**29.09%**	**97.34%**	**8.62%**	**21.03%**	**81.33%**	**1.01%**	**1.73%**	**1.25%**
**Acyclics**																				
RoseoxideII (cis)	1.30	-	-	-	-	-	-	-	-	-	-	-	-	-	-	-	-	-	-	-
RoseoxideI (trans)	1.81	-	0.08	-	0.21	0.26	-	0.03	-	-	-	0.14	0.09	-	-	-	-	-	0.14	0.08
Nerol oxide	3.36	-	1.15	0.11	1.29	7.30	-	0.06	-	-	-	-	-	-	-	-	-	0.21	1.86	2.98
Linalool	278.88	0.13	71.47	0.20	132.88	267.03	0.80	0.07	0.16	0.56	40.54	0.28	0.70	0.23	1.18	1.40	0.15	31.88	208.45	45.29
Hotrienol	1.01	-	0.51	-	1.94	9.17	-	-	-	-	0.11	-	-	-	-	-	-	0.16	0.68	0.61
Neral	47.11	-	17.26	-	9.55	45.11	-	-	0.61	-	9.77	-	2.01	-	-	3.78	-	17.72	53.41	7.51
β-Myrcene	3.14	0.79	3.05	2.07	-	12.13	0.93	1.18	1.32	-	5.08	-	-	2.70	-	1.73	0.86	9.17	27.75	4.67
Geranial	40.42	0.48	30.76	1.08	18.28	62.81	0.45	0.30	1.48	1.52	14.15	1.09	4.49	-	1.51	7.20	-	37.45	108.95	16.22
Citronellol	398.87	3.17	6.27	0.65	19.45	50.57	1.14	1.36	0.17	-	0.68	4.90	2.25	-	5.06	3.10	-	18.03	39.56	5.59
Nerol	813.02	1.80	77.06	2.17	83.06	611.74	0.79	1.21	0.40	6.31	64.84	7.37	4.54	-	4.70	25.83	-	181.17	569.48	302.87
Geraniol	1883.53	5.00	145.33	-	203.33	905.46	6.39	6.49	4.17	10.20	313.27	14.85	10.03	-	5.32	32.29	-	651.52	2426.78	625.07
E-Nerolidol	-	-	-	-	0.42	-	-	-	-	-	0.16	-	-	-	-	-	-	0.18	-	0.39
Geranic acid	3334.14	27.57	2614.39	26.16	2418.64	5815.51	3.29	2.82	50.26	22.95	1279.92	1.72	-	-	13.31	75.14	4.79	3996.61	6040.71	2906.19
SubTOTAL	6806.59	38.94	2967.33	32.44	2889.05	7787.09	13.79	13.52	58.57	41.54	1728.52	30.35	24.11	2.93	31.08	150.47	5.50	4944.10	9477.77	3917.47
**%**	**97.29%**	**90.68%**	**98.94%**	**46.81%**	**97.84%**	**97.99%**	**62.77%**	**36.16%**	**68.05%**	**84.00%**	**98.28%**	**76.60%**	**70.91%**	**2.66%**	**91.38%**	**78.97%**	**18.67%**	**98.99%**	**98.27%**	**98.75%**
TOTAL terpenes	6996.26	42.94	2999.06	69.30	2952.69	7946.92	21.97	37.39	86.07	49.45	1758.83	39.62	34.00	110.22	34.01	190.53	32.18	4994.39	9644.35	3967.09
**%[a]**	**99.93%**	**97.28%**	**99.94%**	**98.79%**	**99.85%**	**99.95%**	**94.66%**	**98.06%**	**98.79%**	**95.22%**	**99.89%**	**93.22%**	**94.92%**	**96.87%**	**93.38%**	**98.65%**	**91.55%**	**99.96%**	**99.97%**	**99.94%**

Table S2. Concentrations (μg/kg) of monoterpenes determined in skin of table grapes. Data are shown as the means (n=3). %[a] indicated that percentage of monoterpenes contents account for the total terpenes contents. - indicated that the compound was not detected. Capital letters refer to the table grape cultivars listed in Figure 1.

Compounds	Cultivars																			
	**A**	**B**	**C**	**D**	**E**	**F**	**G**	**H**	**I**	**J**	**K**	**L**	**M**	**N**	**O**	**P**	**Q**	**R**	**S**	**T**
**Fatty acids derivatives**																				
Hexanal	277.62	39.26	71.45	139.15	150.22	48.50	249.16	103.39	135.58	75.12	90.66	27.46	43.82	46.29	61.18	4.32	146.97	314.54	82.12	131.88
(*Z*)-3-Hexenal	0.39	0.12	-	0.12	0.22	-	0.59	0.24	0.64	0.26	0.02	0.15	-	0.19	0.13	-	0.37	2.19	0.06	-
(*E*)-2-Hexenal	534.42	169.69	79.17	199.95	389.06	99.26	623.62	364.12	193.04	313.78	111.36	238.60	87.26	151.93	294.90	71.41	422.06	432.37	81.73	480.76
Hexanol	53.22	28.12	88.24	318.42	223.00	26.75	36.21	20.64	27.38	81.28	83.31	45.83	45.57	91.46	36.13	67.90	300.87	141.66	85.90	25.61
(*E*)-3-Hexenol	-	-	-	-	-	-	-	-	-	2.72	1.94	-	5.74	2.47	12.79	1.72	5.15	2.14	7.25	1.13
(*Z*)-3-Hexenol	3.65	10.68	19.87	116.70	200.56	13.09	7.16	15.61	37.65	1.56	3.72	15.29	-	11.75	-	21.15	39.70	56.28	-	0.77
(*E*)-2-Hexenol	86.89	43.95	80.16	343.26	265.99	53.38	33.94	26.92	66.42	159.48	137.06	117.28	58.42	47.40	50.97	133.70	425.67	128.49	102.00	61.81
1-Octen-3-ol	1.57	0.26	0.22	0.42	0.36	0.27	0.16	0.19	0.19	1.04	0.40	0.23	0.22	0.55	0.21	0.88	0.22	0.29	0.05	-
Heptanol	-	0.35	0.32	0.46	0.98	0.31	0.33	0.36	0.30	1.07	0.34	0.34	0.32	0.24	0.36	0.47	-	0.29	0.18	0.23
Octanol	1.16	0.58	0.16	0.86	0.44	0.61	-	0.71	0.39	1.17	0.33	0.39	0.85	0.50	0.53	0.93	0.10	0.36	0.28	0.12
Nonanol	0.63	0.45	0.23	0.35	0.42	0.26	0.18	0.25	0.18	2.97	0.22	0.40	0.37	0.14	0.46	0.54	-	-	0.22	0.17
Ethyl acetate	4784.86	2129.60	6.94	20.47	-	35.21	21.48	1358.21	5.92	2823.63	51.70	3823.30	1642.77	21.45	3773.93	3875.26	102.16	-	17.75	13.05
Ethyl Propionate	1.32	-	-	-	-	-	-	0.54	-	0.45	-	0.89	0.44	-	1.23	1.10	-	-	-	-
Propyl acetate	-	0.33	-	-	-	-	-	-	-	0.30	-	0.29	0.05	-	0.34	0.36	-	-	-	-
Ethyl butyrate	69.14	0.37	-	-	-	-	-	-	-	54.91	-	62.18	33.98	-	67.95	82.60	-	-	-	-
Butyl acetate	-	0.05	0.08	0.10	0.15	0.06	0.07	0.04	0.03	0.11	0.06	0.04	0.05	0.22	0.10	0.11	0.06	0.03	0.02	0.07
Ethyl pentanoate	1.89	-	-	-	-	-	-	1.08	-	-	-	0.97	-	-	2.41	2.53	-	-	-	-
Ethyl hexanoate	19.55	4.99	-	2.10	-	-	-	6.71	2.40	12.34	-	23.40	13.14	1.68	18.10	42.47	2.02	-	-	-
Hexyl acetate	-	0.05	-	0.14	0.11	-	0.02	0.01	-	0.11	-	-	0.40	0.02	0.04	1.20	1.36	0.04	-	0.14
(*Z*)-3-Hexenyl acetate	-	-	-	0.45	1.05	-	-	-	0.27	-	-	-	-	-	-	0.63	-	-	-	-
Ethyl heptanoate	0.43	-	-	-	-	-	-	0.09	-	0.52	-	0.50	-	-	0.72	-	-	-	-	-
Hexanoic acid	1.81	1.83	4.09	3.13	3.11	1.61	1.25	0.47	2.31	0.71	1.58	1.72	1.81	0.79	0.72	1.45	1.36	2.69	-	0.68
2-Hexenoic acid	15.32	20.71	44.86	46.39	61.26	20.42	25.10	11.07	29.54	11.51	36.88	13.90	22.98	21.29	15.08	26.07	20.76	34.88	32.11	20.83
Octanoic acid	0.80	0.51	0.57	0.56	0.29	0.32	0.18	0.07	0.03	0.29	0.12	0.33	0.14	0.02	0.67	0.74	0.38	0.11	0.30	0.43
SubTOTAL	17.92	23.05	49.52	50.08	64.66	22.35	26.53	11.60	31.88	12.52	38.57	15.95	24.93	22.10	16.48	28.27	22.49	37.68	32.41	21.94
Pentanal	9.40	-	-	1.14	-	0.87	-	-	-	-	0.57	-	-	-	-	-	-	-	-	-
Octanal	0.96	0.51	0.62	0.72	0.95	0.44	0.31	0.39	0.37	0.39	0.28	0.42	0.44	-	0.30	0.23	0.44	0.56	0.49	0.42
Nonanal	17.20	5.81	6.08	15.70	15.93	13.16	8.37	11.81	10.53	14.74	7.10	12.05	14.81	11.16	10.78	7.69	9.46	11.90	12.45	10.05
**SubTOTAL**	5900.15	2481.27	452.58	1260.67	1378.76	336.87	1034.66	1934.52	545.05	3572.98	566.22	4401.91	1998.51	431.65	4366.51	4373.73	1501.60	1166.50	455.32	770.09
**%**	**90.25%**	**98.18%**	**66.70%**	**98.16%**	**75.58%**	**58.35%**	**98.69%**	**99.32%**	**97.86%**	**97.52%**	**88.31%**	**99.60%**	**99.35%**	**99.36%**	**99.60%**	**99.41%**	**95.51%**	**87.22%**	**49.12%**	**89.79%**
**Isoprenoid derivatives**																				
α-Pinene	-	-	-	-	-	-	-	-	-	-	-	-	-	-	-	-	1.05	0.71	-	-
Phellandrene	0.47	0.17	0.21	-	0.59	0.23	-	0.20	-	0.26	0.17	0.46	0.74	-	0.23	0.44	-	-	0.63	0.25
β-Myrcene	5.50	2.37	-	-	-	-	-	-	-	-	-	-	-	-	-	-	-	2.52	-	-
D-Limonene	42.74	26.55	6.16	0.13	2.27	2.06	-	-	-	-	-	-	-	-	-	-	59.44	44.93	21.34	11.13
γ-Terpinene	3.38	0.89	-	-	-	-	-	0.34	-	0.53	-	1.20	1.61	-	0.59	-	1.10	0.93	-	0.54
P-Cymene	0.39	0.23	0.27	-	-	-	-	0.02	-	0.12	-	0.11	0.41	-	0.07	0.15	0.10	0.03	0.16	0.05
Terpinolene	0.95	0.23	2.30	-	1.88	1.82	-	0.16	-	0.19	0.18	-	0.49	-	0.36	0.45	-	0.39	4.94	1.00
Rose oxide II (cis)	4.85	0.13	-	-	-	-	-	-	-	-	-	-	-	-	-	-	-	-	-	-
Rose oxide I (trans)	1.28	-	-	-	0.60	0.18	-	-	-	-	-	-	-	-	-	-	-	-	-	-
Nerol oxide	-	-	-	-	-	3.76	-	-	-	-	-	-	-	-	-	-	-	-	-	1.12
Linalool	310.40	0.80	123.02	-	328.13	137.82	0.17	-	-	-	2.32	-	-	-	-	-	-	3.14	290.69	10.19
4-Terpineol	4.98	0.35	0.25	-	-	0.25	-	0.54	-	3.12	-	1.19	2.59	-	1.19	1.44	-	-	0.89	0.19
Hotrienol	3.11	-	1.84	-	4.41	11.39	0.04	-	-	-	-	-	-	-	-	-	-	-	2.33	3.77

Neral	3.71	-	-	-	-	0.72	-	-	-	-	1.75	-	-	-	-	-	-	2.20	1.25	0.17
α-Terpineol	36.74	0.61	18.20	0.50	16.11	18.92	0.53	0.52	0.36	0.63	0.91	0.53	0.64	0.28	1.07	1.13	0.32	1.30	39.32	4.15
Geranial	7.90	0.49	0.75	0.24	1.44	1.76	0.47	-	-	-	3.12	0.19	-	-	-	0.47	-	3.64	2.68	0.84
Citronellol	21.91	-	-	-	4.20	1.55	1.21	1.24	1.15	1.95	1.29	1.34	1.20	-	1.51	1.57	-	2.18	1.57	1.18
Nerol	22.51	2.29	8.07	2.44	12.16	12.88	-	2.15	2.10	2.49	7.49	2.27	-	-	-	4.46	-	11.38	17.02	6.05
Geraniol	128.68	3.12	20.50	2.69	20.96	27.29	2.74	2.60	2.42	2.66	35.45	2.75	-	-	2.76	5.68	-	51.91	73.46	23.51
Cedrol	-	0.69	0.87	0.27	1.57	0.47	1.35	1.14	0.85	1.24	0.57	0.86	0.57	0.45	1.02	0.48	0.61	0.41	0.39	0.36
Geranic acid	33.04	1.98	38.52	3.17	22.96	15.78	1.69	1.50	1.15	1.54	16.67	-	-	-	-	1.48	-	43.37	10.92	20.38
β-Damascenone	0.01	0.01	0.11	0.07	0.01	0.04	0.14	0.17	0.01	0.09	0.02	0.03	0.05	0.01	0.18	0.01	0.01	0.01	0.04	0.07
Geranyl acetone	-	-	-	-	-	-	-	-	-	-	-	0.34	0.19	0.07	0.17	0.08	0.14	0.28	0.35	0.12
β-Ionone	0.10	-	-	0.08	0.17	0.05	0.08	0.07	0.06	0.07	-	0.07	0.06	0.06	0.08	-	0.07	0.07	0.06	0.06
**SubTOTAL**	632.64	41.33	221.51	10.36	418.66	237.25	8.54	10.75	8.78	14.88	70.00	11.34	8.92	0.86	9.43	17.85	62.85	169.47	468.74	85.26
**%**	**9.68%**	**1.64%**	**32.65%**	**0.81%**	**22.95%**	**41.10%**	**0.81%**	**0.55%**	**1.58%**	**0.41%**	**10.92%**	**0.26%**	**0.44%**	**0.20%**	**0.22%**	**0.41%**	**4.00%**	**12.67%**	**50.57%**	**9.94%**
**Amino acids derivatives**																				
2-Ethyl hexanol	1.67	1.31	1.92	1.76	3.79	1.18	1.23	1.19	2.16	1.56	1.56	1.39	1.55	1.40	1.63	0.90	1.26	0.96	1.58	1.04
Phenylethyl alcohol	1.23	1.69	0.20	0.28	0.19	-	-	0.29	0.03	60.63	1.19	1.27	0.19	-	0.43	2.46	0.45	0.08	0.04	-
Ethyl isobutyrate	-	-	-	-	-	-	-	-	-	-	-	0.34	0.18	-	0.38	0.35	-	-	-	-
Ethyl 3-methylbutanoate	-	-	-	-	-	-	-	-	-	0.11	-	-	-	-	-	0.17	-	-	-	-
Ethyl 3-hydroxybutyrate	1.13	-	-	-	-	-	-	-	-	2.65	-	0.67	-	-	-	0.54	-	-	-	-
Methyl salicylate	0.23	0.26	-	0.40	1.19	0.05	1.93	0.21	0.13	0.15	-	0.07	0.69	0.02	4.20	0.74	0.41	0.30	0.40	0.62
2-Methylbutanal	-	-	0.24	0.35	1.13	0.14	0.11	-	0.12	-	0.11	-	-	-	-	-	0.42	-	0.17	-
3-Methylbutanal	-	-	0.47	0.67	5.30	0.28	0.27	-	0.18	-	0.22	-	-	0.08	-	-	0.98	-	0.28	-
Benzaldehyde	0.42	0.49	1.16	8.90	11.14	1.37	1.33	0.10	0.50	6.78	1.64	0.88	0.74	0.30	0.74	2.48	0.45	-	-	0.48
Phenylacetaldehyde	-	0.93	0.44	0.86	4.12	0.16	0.36	0.65	-	3.93	0.23	1.51	0.76	0.12	0.51	0.28	3.85	0.07	0.35	0.14
**SubTOTAL**	4.68	4.68	4.43	13.22	26.86	3.18	5.23	2.44	3.12	75.81	4.95	6.13	4.11	1.92	7.89	7.92	7.82	1.41	2.82	2.28
**%**	**0.07%**	**0.19%**	**0.65%**	**1.03%**	**1.47%**	**0.55%**	**0.50%**	**0.13%**	**0.56%**	**2.07%**	**0.77%**	**0.14%**	**0.20%**	**0.44%**	**0.18%**	**0.18%**	**0.50%**	**0.11%**	**0.30%**	**0.27%**

Table S3. Concentrations (μg/kg) of volatile compounds from different synthesis pathwaysd determined in pulp juice of table grapes. Data are shown as the means (n=3). - indicated that the compound was not detected. Capital letters refer to the table grape cultivars listed in Figure 1.

Compounds	Cultivars																			
	**A**	**B**	**C**	**D**	**E**	**F**	**G**	**H**	**I**	**J**	**K**	**L**	**M**	**N**	**O**	**P**	**Q**	**R**	**S**	**T**
**Fatty acids derivatives**																				
Hexanal	504.30	93.18	78.18	134.57	314.69	174.58	198.05	117.08	88.41	207.41	106.19	235.38	116.34	143.64	140.32	185.85	198.34	186.25	267.30	131.10
(*Z*)-3-Hexenal	1.06	0.24	0.05	0.47	1.19	-	0.76	0.39	0.38	0.14	-	0.65	0.40	0.79	0.31	0.54	0.65	0.73	0.23	0.13
(*E*)-2-Hexenal	976.35	207.02	232.73	338.70	790.85	508.46	457.79	310.21	216.75	393.15	107.89	508.18	341.91	459.53	405.24	473.56	445.87	435.66	514.55	389.09
Hexanol	13.22	104.17	18.46	64.31	59.65	18.26	44.56	45.52	117.83	177.41	226.16	58.81	42.36	82.63	16.22	74.09	93.85	30.20	32.13	8.64
(*E*)-3-Hexenol	-	2.71	-	1.08	-	-	-	-	1.93	3.62	5.94	-	-	-	-	-	1.71	-	-	-
(*Z*)-3-Hexenol	4.28	4.11	1.69	23.43	41.87	4.31	1.54	10.00	8.81	2.29	1.13	11.43	6.23	7.05	5.16	16.18	19.24	13.28	6.69	0.61
(*E*)-2-Hexenol	52.99	239.15	25.81	61.16	58.93	39.23	63.05	54.30	263.49	240.87	108.55	107.41	75.19	52.43	25.79	98.07	143.52	46.75	55.85	26.03
2-Heptanol	-	-	-	0.26	-	-	0.33	0.29	-	-	-	-	-	-	-	-	-	-	-	-
1-Octen-3-ol	0.36	0.47	0.34	0.54	0.71	0.33	0.42	0.35	0.35	1.67	0.88	2.10	0.60	0.74	0.99	5.05	0.30	0.43	0.21	0.21
Heptanol	0.40	0.69	0.30	0.57	0.65	-	0.39	0.40	0.33	2.97	0.51	1.25	0.62	0.75	0.62	1.31	0.53	0.54	0.71	0.32
Octanol	1.69	0.77	0.19	0.97	0.70	0.56	0.58	0.49	0.40	1.26	0.16	1.69	0.90	0.72	1.67	2.60	0.31	0.93	0.87	0.34
Nonanol	1.49	0.58	0.13	0.78	0.37	0.47	0.39	0.45	0.35	1.42	0.45	1.70	0.73	0.36	2.70	2.15	0.16	0.70	1.17	0.20
Ethyl acetate	850.97	134.41	4.36	2.59	15.22	7.11	7.03	82.14	3.54	1949.87	575.90	1245.11	103.45	72.75	1746.41	1242.30	1.99	10.20	6.79	7.34
Ethyl Propionate	-	-	-	-	-	-	-	-	-	0.26	-	0.09	-	-	0.15	0.32	-	-	-	-
Propyl acetate	-	-	-	-	-	-	-	-	-	0.11	-	-	-	-	0.02	0.09	-	-	-	-
Ethyl butyrate	3.92	-	-	-	-	-	-	1.41	-	21.97	-	8.62	1.36	-	4.96	18.63	-	-	-	-
Butyl acetate	-	0.04	0.05	0.11	0.16	0.06	-	0.03	0.02	0.12	0.27	0.09	0.04	0.45	0.05	0.09	0.07	0.04	0.06	-
Ethyl hexanoate	15.07	1.70	-	1.75	-	-	1.60	2.66	1.56	25.81	-	10.52	4.32	4.50	7.36	19.48	3.26	-	-	-
Hexyl acetate	-	-	0.22	0.02	0.19	-	-	0.24	-	0.10	1.30	-	-	0.37	-	0.36	0.11	-	-	-
Ethyl heptanoate	-	-	-	-	-	-	-	-	-	0.79	-	-	-	-	0.44	-	-	-	-	-
Ethyl octanoate	2.77	-	-	-	-	-	-	-	-	1.10	-	0.83	-	-	-	-	-	-	-	-
Hexanoic acid	-	0.20	-	2.04	-	-	-	-	0.85	-	-	-	0.84	1.14	-	-	1.21	-	-	-
2-Hexenoic acid	-	20.71	44.14	39.89	77.58	88.58	-	26.62	-	-	24.84	-	56.29	-	30.71	41.32	25.61	30.28	56.32	36.58
Octanoic acid	-	-	-	0.32	-	-	-	-	-	0.15	0.89	-	-	-	-	0.71	-	-	-	-
Pentanal	4.24	1.59	0.45	0.81	1.80	1.09	1.06	1.32	-	0.56	-	2.40	1.28	-	0.95	-	1.39	0.37	2.57	1.32
Octanal	0.53	0.19	0.26	0.29	1.02	0.52	0.87	0.37	0.22	0.68	0.25	0.62	0.25	1.67	0.23	0.47	0.76	0.31	0.66	0.37
Nonanal	-	1.13	2.02	0.73	4.07	8.99	1.62	1.14	0.95	3.17	0.68	1.87	1.19	4.61	3.77	1.49	1.97	3.05	-	4.80
(*E*)-2-Octenal	-	-	0.15	0.19	0.57	0.19	-	-	-	-	-	0.14	-	-	-	0.21	-	-	0.20	0.19
**SubTOTAL**	2433.64	813.06	409.53	675.58	1370.22	852.74	780.04	655.41	706.17	3036.90	1161.99	2198.89	754.30	834.13	2394.07	2184.87	940.85	759.72	946.31	607.27
**%**	**25.74%**	**91.99%**	**11.95%**	**89.28%**	**31.04%**	**9.67%**	**94.45%**	**92.48%**	**86.52%**	**96.09%**	**39.60%**	**96.24%**	**89.75%**	**87.01%**	**94.62%**	**90.41%**	**96.16%**	**13.19%**	**8.92%**	**13.21%**
**Isoprenoid derivatives**																				
α-Pinene	-	-	-	-	-	-	-	-	0.24	-	-	-	-	1.25	-	0.13	-	-	-	-
β-Pinene	19.79	-	-	-	3.68	-	-	-	-	0.21	-	0.11	0.14	1.55	-	-	0.34	-	-	3.68
Phellandrene	6.96	0.15	0.39	0.06	0.77	1.87	-	-	-	0.22	1.17	0.28	0.25	0.16	0.20	0.45	-	0.56	1.89	0.58
β-Myrcene	3.14	0.79	3.05	2.07	-	12.13	0.93	1.18	1.32	-	5.08	-	-	2.70	-	1.73	0.86	9.17	27.75	4.67
D-Limonene	42.63	2.43	3.38	34.55	16.12	14.98	7.32	21.96	25.70	1.23	4.95	6.03	4.20	100.27	0.62	21.95	21.35	32.61	40.92	7.79
γ-Terpinene	4.33	0.18	0.98	0.93	1.02	2.38	0.29	0.69	0.68	1.46	0.90	-	0.44	2.77	-	2.49	0.58	0.97	4.50	0.86
P-Cymene	1.82	0.17	0.40	0.29	0.77	0.90	0.18	0.24	0.21	0.44	0.84	0.31	0.27	0.79	0.21	0.60	0.30	0.47	0.96	0.35
Terpinolene	11.67	0.45	3.44	0.45	5.29	16.02	0.13	0.44	0.25	1.09	2.36	0.83	0.93	0.50	0.74	3.07	0.27	2.42	13.48	4.27
Rose oxide II (cis)	1.30	-	-	-	-	-	-	-	-	-	-	-	-	-	-	-	-	-	-	-
Rose oxide I (trans)	1.81	-	0.08	-	0.21	0.26	-	0.03	-	-	-	0.14	0.09	-	-	-	-	-	0.14	0.08
Nerol oxide	3.36	-	1.15	0.11	1.29	7.30	-	0.06	-	-	-	-	-	-	-	-	-	0.21	1.86	2.98
Linalool	278.88	0.13	71.47	0.20	132.88	267.03	0.80	0.07	0.16	0.56	40.54	0.28	0.70	0.23	1.18	1.40	0.15	31.88	208.45	45.29
4-Terpineol	4.40	0.40	0.56	-	0.29	1.32	-	-	-	1.84	0.34	1.15	1.78	-	1.08	2.44	-	-	0.87	0.66

Hotrienol	1.01	-	0.51	-	1.94	9.17	-	-	-	-	0.11	-	-	-	-	-	-	0.16	0.68	0.61
Neral	47.11	-	17.26	-	9.55	45.11	-	-	0.61	-	9.77	-	2.01	-	-	3.78	-	17.72	53.41	7.51
α-Terpineol	98.07	0.22	22.58	0.42	35.70	122.36	0.26	0.54	0.42	1.42	19.75	0.56	1.77	-	0.08	8.67	1.12	13.26	103.96	31.43
Geranial	40.42	0.48	30.76	1.08	18.28	62.81	0.45	0.30	1.48	1.52	14.15	1.09	4.49	-	1.51	7.20	-	37.45	108.95	16.22
Citronellol	398.87	3.17	6.27	0.65	19.45	50.57	1.14	1.36	0.17	-	0.68	4.90	2.25	-	5.06	3.10	-	18.03	39.56	5.59
Myrtenol	-	-	-	0.16	-	-	-	-	-	-	-	-	0.11	-	-	0.26	-	-	-	-
Nerol	813.02	1.80	77.06	2.17	83.06	611.74	0.79	1.21	0.40	6.31	64.84	7.37	4.54	-	4.70	25.83	-	181.17	569.48	302.87
Geraniol	1883.53	5.00	145.33	-	203.33	905.46	6.39	6.49	4.17	10.20	313.27	14.85	10.03	-	5.32	32.29	-	651.52	2426.78	625.07
E-Nerolidol	-	-	-	-	0.42	-	-	-	-	-	0.16	-	-	-	-	-	-	0.18	-	0.39
Cedrol	5.10	1.12	1.81	1.38	4.37	3.84	1.50	1.30	1.47	1.99	1.88	2.68	1.33	3.80	2.45	1.77	2.42	1.86	2.75	2.23
Geranic acid	3334.14	27.57	2614.39	26.16	2418.64	5815.51	3.29	2.82	50.26	22.95	1279.92	1.72	-	-	13.31	75.14	4.79	3996.61	6040.71	2906.19
β-Damascenone	0.02	0.01	0.05	0.03	0.04	0.08	0.07	0.01	0.01	0.14	0.10	0.02	0.03	0.06	0.04	0.06	0.04	0.01	0.05	0.03
Geranyl acetone	-	0.07	0.24	0.12	0.48	-	0.12	0.12	0.10	0.19	-	0.12	0.12	0.28	0.17	0.14	0.19	-	-	-
β-Ionone	0.46	0.20	0.18	0.38	0.57	0.40	0.24	0.26	0.26	0.36	0.38	0.41	0.32	0.46	0.44	0.32	0.54	0.33	0.46	0.27
**SubTOTAL**	7001.86	44.43	3001.35	70.68	2958.17	7951.61	23.64	38.52	87.50	52.62	1761.18	43.05	36.29	114.58	37.07	193.65	26.89	4996.61	9647.61	3969.59
%	**74.04%**	**5.03%**	**87.58%**	**9.34%**	**67.02%**	**90.15%**	**2.86%**	**5.44%**	**10.72%**	**1.67%**	**60.02%**	**1.88%**	**4.32%**	**11.95%**	**1.47%**	**8.01%**	**2.75%**	**86.77%**	**90.97%**	**86.33%**
**Amino acids derivatives**																				
2-Ethyl hexanol	1.60	1.37	1.23	2.88	6.20	1.33	1.88	1.54	5.54	2.60	4.51	2.05	2.22	5.98	1.40	3.74	2.75	1.46	2.28	1.22
Benzyl alcohol	-	-	-	-	1.01	0.04	0.08	0.05	-	-	0.06	-	-	-	-	-	-	-	-	4.08
Phenylethyl alcohol	-	7.49	-	-	-	-	-	-	-	28.52	-	22.47	-	-	-	-	-	-	-	-
Ethyl isobutyrate	-	-	-	-	-	-	-	-	-	0.35	-	-	-	-	0.02	0.24	-	-	-	-
Ethyl 3-methylbutanoate	-	-	-	-	-	-	-	-	-	-	-	-	-	-	-	0.07	-	-	-	-
Methyl salicylate	7.08	2.29	0.25	0.66	3.38	1.38	1.69	0.32	0.23	0.52	-	2.11	7.54	0.87	45.00	1.50	1.39	0.43	-	0.42
3-Methylbutanal	0.38	0.23	0.32	0.15	1.06	0.37	-	0.06	0.02	1.04	-	0.33	0.27	-	-	-	0.48	-	-	0.05
Benzaldehyde	10.40	13.89	13.79	6.50	71.98	13.04	18.52	12.25	16.69	35.55	6.35	14.66	38.48	3.09	52.72	32.29	3.99	-	8.48	15.16
Phenylacetaldehyde	1.45	1.12	0.36	0.27	1.91	0.35	-	0.56	-	2.24	-	1.35	1.31	-	-	0.20	2.04	-	0.38	0.46
**SubTOTAL**	20.91	26.39	15.95	10.46	85.54	16.51	22.17	14.78	22.48	70.82	10.92	42.97	49.82	9.94	99.14	38.04	10.65	1.89	11.14	21.39
%	**0.22%**	**2.99%**	**0.47%**	**1.38%**	**1.94%**	**0.19%**	**2.68%**	**2.09%**	**2.75%**	**2.24%**	**0.37%**	**1.88%**	**5.93%**	**1.04%**	**3.92%**	**1.57%**	**1.09%**	**0.03%**	**0.11%**	**0.47%**

Table S4. Concentrations (μg/kg) of volatile compounds from different synthesis pathwaysd determined in skin of table grapes. Data are shown as the means (n=3). - indicated that the compound was not detected. Capital letters refer to the table grape cultivars listed in Figure 1.

**Compounds**	Pulp juice		Skin	
	Calibration graphs	**r^2**	Calibration graphs	**r^2**
**(A) C_6 alcohols**				
Hexanal	y = 4.5912x - 0.1010	0.9975	y = 3.1725x - 0.0382	0.9970
(*Z*)-3-Hexenal	y = 1.8035x - 0.0040	0.9992	y = 2.5281x - 0.0111	0.9958
(*E*)-2-Hexenal	y = 4.5531x - 0.0035	0.9994	y = 4.6736x - 0.0050	0.9990
Hexanol	y = 9.2890x - 0.1199	0.9965	y = 7.8984x - 0.0400	0.9974
(*Z*)-3-Hexenol	y = 11.0240x - 0.0027	0.9853	y = 17.0820x - 0.0051	0.9989
(*E*)-2-Hexenol	y = 13.9480x - 0.0047	0.9994	y = 13.7670x - 0.0034	0.9997
**(B) Alcohols**				
1-Octen-3-ol	y = 1.3250x - 0.0069	0.9980	y = 1.6180x - 0.0134	0.9986
Heptanol	y = 1.1881x - 0.0076	0.9970	y = 1.3041x - 0.0091	0.9960
Octanol	y = 0.9754x - 0.0027	0.9928	y = 1.0166x - 0.0015	0.9952
Benzyl alcohol	y = 1.3950x - 0.0047	0.9994	y = 0.9843x + 0.0052	0.9994
Phenylethyl alcohol	y = 0.6585x - 0.0074	0.9912	y = 0.7681x + 0.0097	0.9987
**(C) Esters**				
Ethyl acetate	y = 33.1230x - 0.1999	0.9981	y = 35.3580x - 0.2197	0.9980
Ethyl isobutyrate	y = 6.4466x - 0.0788	0.9910	y = 7.2902x - 0.0932	0.9992
Ethyl butyrate	y = 3.4627x - 0.0602	0.9818	y = 2.4866x - 0.0293	0.9996
Butyl acetate	y = 0.7849x - 0.0009	0.9998	y = 0.6986x +0.0022	0.9998
Ethyl pentanoate	y =12.8954x+ 0.0218	0.9980	y = 10.6769x - 0.0016	0.9954
Ethyl hexanoate	y = 0.9127x + 0.0215	0.9983	y = 0.9278x + 0.0284	0.9964
Hexyl acetate	y = 0.9114x - 0.0003	0.9990	y = 0.8568x - 0.0018	0.9954
Methyl salicylate	y = 3.8718x - 0.0023	0.9862	y = 3.1641x - 0.0009	0.9943
**(D) Acids**				
Hexanoic acid	y = 19.2880x - 0.0240	0.9990	y = 25.0740x - 0.0336	0.9976
2-Hexenoic acid	y = 93.9740x - 0.0108	0.9912	y = 79.4640x - 0.8541	0.9984
Octanoic acid	y = 9.0389x - 0.0052	0.9714	y = 14.6190x - 0.0064	0.9812
**(E)Aldehydes**				
3-Methylbutanal	y = 17.4890x - 0.0137	0.9959	y = 11.6450x - 0.0062	0.9995
Octanal	y = 1.4606x - 0.0019	0.9965	y = 1.5628x - 0.0029	0.9997
Pentanal	y = 1.8075x - 0.0040	0.9967	y = 2.5201x - 0.0111	0.9975
Nonanal	y = 3.9123x - 0.0087	0.9950	y = 3.3018x - 0.0104	0.9952
Benzaldehyde	y = 7.3396x - 0.0116	0.9988	y = 5.6839x + 0.0011	0.9924
**(F) Terpenes**				
Phellandrene	y = 1.7816x + 0.0012	0.9977	y = 1.6636x + 0.0014	0.9953
β-Myrcene	y = 1.4759x + 0.0083	0.9989	y = 1.1303x + 0.0216	0.9994
D-Limonene	y = 1.6730x - 0.0056	0.9994	y = 1.3342x - 0.0110	0.9990
P-Cymene	y = 0.8372x - 0.0021	0.9999	y = 0.5940x + 0.0015	0.9988
Terpinolene	y = 1.1842x + 0.0008	0.9990	y = 0.9242x + 0.0059	0.9974
Rose oxide II (cis)	y = 0.8258x - 0.0640	0.9984	y = 0.7654x - 0.0011	0.9990
Rose oxide I (trans)	y = 0.8469x - 0.0870	0.9978	y = 0.7236x - 0.0347	0.9980
Linalool	y = 1.0045x - 0.0052	0.9995	y = 0.9860x - 0.0042	0.9993
4-Terpineol	y = 1.7880x - 0.0003	0.9995	y = 1.8654x - 0.0005	0.9993
Neral	y = 4.7844x - 0.0034	0.9996	y = 7.7458x - 0.0035	0.9987
α-Terpineol	y = 2.2722x + 0.0023	0.9978	y = 2.9073x - 0.0149	0.9959
Geranial	y = 5.5101x + 0.0018	0.9976	y = 9.0050x - 0.01020	0.9980
Citronellol	y = 1.4392x + 0.0119	0.9975	y = 2.0652x - 0.0249	0.9987
Nerol	y = 2.6263x - 0.0117	0.9927	y = 1.8067x - 0.0045	0.9997
Geraniol	y = 4.4518x + 0.0223	0.9961	y = 7.9694x - 0.0197	0.9957
Geranic acid	y = 48.1500x + 0.0086	0.9962	y = 124.5300x - 0.1318	0.9971
**(G) C_{13}-Norisoprenoids**				
β-Damascenone	y = 0.8996x + 0.0003	0.9982	y = 0.7654x + 0.0002	0.9994
Geranyl acetone	y = 0.6941x - 0.0008	0.9923	y = 0.5892x + 0.0006	0.9965
β-Ionone	y = 0.4747x + 0.0005	0.9983	y = 0.5041x + 0.0021	0.9981

Table S5. Quantitative standards and calibration graphs for quantification of volatile compounds in pulp juice and skin of table grape. y, concentration ratio of a compound to the 2-octanol (internal standard); x, peak area ratio of a compound to the 2-octanol. r, regression coefficient.

Compounds	Pulp juice				Skin			
	Concentration in (μg/kg)	Added (μg/L)	Recovery (%)	Reproducibility r.s.d. (%) (n=4)	Concentration in (μg/kg)	Added (μg/L)	Recovery (%)	Reproducibility r.s.d. (%) (n=4)
**(A) C_6 alcohols**								
Hexanal	33.06 ±1.94	203.34	71.25 ±0.39	0.39	97.61 ±9.05	720.32	97.6 ±1.14	2.78
		101.67	83.74 ±12.73	8.73		200.00	80.11 ±1.69	1.55
		20.33	100.67 ±0.13	0.13		72.02	84.98 ±4.25	4.66
(*Z*)-3-Hexenal	0.00	3.15	82.79 ±11.63	11.63	0.00	2.02	86.23 ±0.18	4.21
		1.57	80.76 ±8.72	8.72		1.01	65.42 ±0.11	7.66
		0.32	69.64 ±9.10	9.10		0.20	76.21 ±0.04	1.28
(*E*)-2-Hexenal	8.85 ±1.44	648.70	84.80 ±0.42	0.42	245.48 ±52.62	806.75	82.93 ±5.81	2.78
		324.34	99.61 ±4.99	4.99		403.38	95.29 ±9.22	5.61
		64.87	93.85 ±3.85	3.85		80.68	112.52 ±14.66	7.80
Hexanol	18.68 ±1.88	340.84	120.80 ±2.34	2.34	187.94 ±15.39	331.62	74.97 ±1.44	0.45
		170.42	121.33 ±10.14	10.14		165.81	85.55 ±2.94	3.46
		34.08	147.78 ±4.24	4.24		33.16	97.67 ±3.32	1.92
(*E*)-3-Hexenol	0.00	10.73	87.21 ±2.15	2.15	4.34 ±0.61	48.12	67.02 ±15.28	4.33
		5.36	116.34 ±7.76	7.76		24.07	59.82 ±21.11	12.94
		1.07	294.33 ±3.33	3.33		4.81	21.95 ±26.77	32.84
(*E*)-2-Hexenol	5.11 ±0.38	450.28	104.32 ±1.56	1.56	42.91 ± 4.67	580.23	71.89 ±3.14	4.12
		225.14	119.79 ±6.35	6.35		290.16	82.28 ±6.10	1.91
		45.03	113.22 ±1.24	1.24		58.02	96.02 ±13.68	4.15
**(B) Alcohols**								
1-Octen-3-ol	0.00	1.57	98.37 ±11.22	11.22	0.00	7.87	92.25 ±6.47	8.36
		0.79	101.93 ±6.79	6.79		3.94	93.54 ±5.89	1.54
		0.16	99.81 ±7.82	7.82		0.78	92.47 ±8.17	3.66
Heptanol	0.00	1.07	72.31 ±8.37	8.37	1.52 ±0.22	5.17	76.68 ±10.31	5.20
		0.53	75.46 ±12.05	12.05		2.59	73.49 ±9.64	8.13
		0.11	77.94 ±6.39	6.39		0.52	87.42 ±9.52	7.84
Octanol	0.78 ±0.1	1.89	159.94 ±21.82	11.82	0.09 ±0.02	4.99	78.42 ±5.16	3.85
		0.94	205.04 ±71.41	11.41		2.49	75.91 ±2.91	7.56
		0.19	122.02 ±43.76	3.76		0.50	83.16 ±6.14	6.57
Benzyl alcohol	0.00	4.54	69.09 ±8.71	8.71	0.00	8.34	69.12 ±1.56	1.67
		2.27	72.60 ±6.66	6.66		4.17	71.64 ±1.89	2.88
		0.45	77.40 ±6.81	6.81		0.83	76.27 ±1.04	0.96
Phenylethyl alcohol	0.00	8.76	83.31 ±7.89	7.89	0.00	66.38	112.23 ±3.68	1.99
		4.38	86.97 ±8.53	8.53		33.19	96.62 ±5.31	0.95
		0.88	104.29 ±8.15	8.15		6.34	88.48 ±4.45	2.67
**(C) Esters**								
Ethyl acetate	21.38 ±5.29	1937.50	76.31 ±1.02	1.02	216.72 ±18.01	1484.17	83.83 ±8.45	4.34
		968.25	83.86 ±7.72	7.72		742.09	89.89 ±3.12	7.18
		1.94	111.71 ±6.96	6.96		148.42	86.00 ±1.40	2.03
Ethyl isobutyrate	0.00	1.84	83.13 ±3.49	3.49	0.00	1.16	76.15 ±10.32	4.57
		0.92	97.35 ±4.78	4.78		0.51	85.78 ±5.49	8.21
		0.18	105.40 ±6.34	6.34		0.12	93.79 ±9.19	11.73
Ethyl butyrate	0.53 ±0.09	49.50	87.24 ±3.09	3.09	0.47 ±0.03	47.51	106.82 ±5.64	5.59
		24.75	87.12 ±10.71	10.71		23.76	78.47 ±3.21	2.86
		4.95	111.00 ±3.28	3.28		4.75	97.69 ±5.59	4.79
Butyl acetate	0.00	1.87	82.71 ±7.94	7.94	0.00	2.34	119.21 ±12.36	11.66
		0.93	101.75 ±22.33	22.33		1.17	135.46 ±14.58	8.75
		0.19	122.12 ±17.41	17.41		0.23	108.76 ±8.76	9.67
Ethyl pentanoate	0.00	2.69	110.10 ±14.39	14.39	0.00	8.07	127.00 ±35.49	22.94
		1.34	82.73 ±6.71	6.71		4.03	124.26 ±23.12	9.45
		0.27	65.39 ±9.17	9.17		0.81	109.47 ±14.21	12.84
Ethyl hexanoate	1.98 ±0.54	27.30	115.50 ±6.88	6.88	2.13 ±0.38	27.30	127.65 ±8.68	1.98
		13.65	116.14 ±3.41	3.41		13.65	139.81 ±15.22	0.63
		2.73	102.81 ±2.43	2.43		2.73	127.87 ±30.79	14.54
Hexyl acetate	1.08 ±0.23	1.38	109.41 ±13.85	13.85	1.78 ±0.09	1.24	114.90 ±7.54	3.10
		0.69	137.38 ±10.31	10.31		0.62	114.19 ±11.83	1.14
		0.14	109.90 ±26.54	6.54		0.12	108.73 ±11.18	10.22
Methyl salicylate	2.54 ±0.17	7.20	70.63 ±11.74	11.74	0.00	54.73	76.17 ±4.73	1.83
		3.60	75.44 ±7.89	7.89		27.37	72.38 ±2.19	1.76
		0.72	74.37 ±6.49	6.49		5.47	88.74 ±2.37	1.00
**(D) Acids**								
Hexanoic acid	0.00	4.96	18.77 ±6.24	6.24	0.00	7.60	38.14 ±0.10	16.79
		2.48	0.00			3.81	0.00	
		0.50	0.00			0.76	0.00	
2-Hexenoic acid	0.00	64.55	33.41 ±9.17	9.17	16.53 ±3.12	20.89	17.47 ±3.31	6.77
		32.27	38.71 ±12.87	12.87		10.45	12.76 ±4.62	11.13
		0.65	0.00			2.09	23.40 ±27.51	16.24
Octanoic acid	0.00	2.51	0.00		2.13 ±0.30	2.21	28.86 ±19.81	9.12
		2.25	0.00			1.11	38.65 ±14.75	27.70
		0.25	0.00			0.22	56.43 ±68.53	9.05
**(E)Aldehydes**								
3-Methylbutanal	0.00	5.57	79.61 ±16.56	16.56	0.00	2.96	75.62 ±11.37	2.65
		2.78	95.83 ±10.22	10.22		1.48	74.78 ±8.79	11.67
		0.56	64.39 ±11.63	11.63		0.30	77.42 ±21.56	8.27
Octanal	0.32 ±0.16	2.00	107.52 ±6.12	6.12	0.21 ±0.01	2.00	102.26 ±7.54	1.26
		1.00	132.12 ±7.98	7.98		1.00	119.54 ±20.66	8.48
		0.20	114.01 ±30.46	3.46		0.20	121.76 ±25.11	4.56

Compounds	Pulp juice				Skin			
	Concentration in (μg/kg)	Added (μg/L)	Recovery (%)	Reproducibility r.s.d. (%) (n=4)	Concentration in (μg/kg)	Added (μg/L)	Recovery (%)	Reproducibility r.s.d. (%) (n=4)
Pentanal	0.00	5.14	96.47 ±2.43	2.43	0.00	3.88	51.36 ±22.70	0.07
		2.57	108.46 ±5.68	5.68		1.94	58.16 ±11.26	5.08
		0.51	87.62 ±7.32	7.32		0.39	72.79 ±53.68	10.85
Nonanal	6.24 ±0.58	13.79	111.08 ±2.24	2.24	4.35 ±0.61	14.75	90.18 ±1.64	0.07
		6.89	121.02 ±14.07	14.07		7.38	88.92 ±3.90	5.08
		1.38	86.85 ±9.53	9.53		1.48	91.44 ±7.78	10.85
Benzaldehyde	2.36 ±0.46	15.30	108.03 ±2.58	2.58	1.25 ±0.08	75.62	88.71 ±4.75	3.43
		7.65	104.68 ±3.42	3.42		37.81	89.81 ±7.50	4.56
		1.53	103.18 ±5.18	5.18		7.56	98.30 ±17.44	13.20
**(F) Terpenes**								
Phellandrene	0.11 ±0.01	2.12	88.34 ±10.4	10.40	0.08 ±0.01	10.69	118.55 ±12.71	2.19
		1.06	93.01 ±3.40	3.40		5.35	130.03 ±22.55	5.12
		0.21	88.57 ±4.83	4.83		1.07	167.29 ±35.92	10.46
β-Myrcene	0.98 ±0.36	12.50	170.10 ±22.96	22.96	2.59 ±0.22	25.00	140.14 ±11.22	3.56
		6.25	147.43 ±5.82	5.82		12.50	136.29 ±15.41	4.49
		1.25	121.54 ±10.98	10.98		2.50	118.59 ±20.68	8.07
D-Limonene	4.24 ±0.40	45.16	108.72 ±12.91	0.91	4.37 ±0.31	106.90	123.64 ±14.55	1.73
		22.58	101.57 ±11.44	11.44		53.45	116.90 ±22.88	4.18
		4.52	106.95 ±2.49	2.49		10.69	132.91 ±31.09	6.41
P-Cymene	0.00	2.15	175.21 ±18.96	18.96	0.33 ±0.01	3.15	115.83 ±9.13	1.74
		1.08	185.43 ±23.37	3.37		1.58	119.81 ±14.73	6.60
		0.22	168.91 ±17.17	7.17		0.32	121.14 ±20.49	18.06
Terpinolene	0.54 ±0.01	8.40	103.58 ±3.42	3.42	5.74 ±0.43	18.40	68.56 ±10.35	6.54
		4.20	104.66 ±1.87	1.87		9.20	78.57 ±5.47	7.50
		0.84	98.79 ±7.21	7.21		1.84	89.54 ±16.47	1.54
Rose oxide	0.00	6.00	72.05 ±2.64	2.64	0.00	5.74	77.46 ±21.33	6.25
		3.00	122.75 ±0.33	0.33		2.87	134.04 ±66.77	11.25
		0.60	100.72 ±0.39	0.39		0.57	102.22 ±19.93	22.28
Linalool	49.78 ±2.24	78.88	128.81 ±1.21	1.21	109.09 ±5.26	358.42	113.30 ±5.19	3.01
		39.44	125.81 ±1.54	1.54		179.21	110.13 ±7.75	7.63
		7.89	110.42 ±1.40	1.40		35.84	104.75 ±7.34	6.92
4-Terpineol	1.04 ±0.09	3.86	101.90 ±0.84	0.84	0.48 ±0.03	3.46	100.26 ±5.35	2.95
		1.93	117.34 ±1.67	1.67		1.73	100.36 ±7.89	3.01
		0.39	211.44 ±1.13	1.13		0.35	104.72 ±14.00	1.72
Neral	0.00	62.91	78.89 ±9.06	9.06	0.00	4.62	129.47 ±7.34	8.23
		31.45	84.18 ±7.13	7.13		2.31	131.03 ±10.36	3.44
		6.29	69.48 ±5.34	5.34		0.46	70.14 ±2.89	5.07
α-Terpineol	3.59 ±0.61	56.20	78.06 ±1.28	1.28	41.88 ±3.66	219.61	115.48 ±6.23	3.88
		28.10	82.34 ±3.12	3.12		109.81	112.35 ±9.50	8.96
		5.62	88.51 ±2.01	2.01		21.96	104.67 ±10.34	10.23
Geranial	0.11 ±0.05	10.85	85.56 ±1.49	1.49	0.94 ±0.08	201.99	102.93 ±7.89	5.05
		5.42	94.12 ±0.31	0.31		100.99	107.73 ±11.61	2.46
		1.09	112.45 ±2.07	2.07		20.20	115.48 ±23.36	9.18
Citronellol	1.10 ±0.09	16.98	69.84 ±0.84	0.84	1.13 ±0.09	482.36	115.76 ±3.37	3.27
		8.49	110.19 ±1.54	1.54		241.18	113.59 ±30.38	11.27
		1.70	88.36 ±3.86	3.86		48.24	79.76 ±64.96	3.66
Nerol	0.00	89.47	93.31 ±1.00	1.00	0.00	25.86	97.69 ±5.39	3.88
		44.73	101.78 ±3.99	3.99		12.93	99.79 ±5.65	10.45
		8.95	103.61 ±3.51	3.51		2.59	94.28 ±4.78	6.72
Geraniol	4.59 ±0.33	54.31	117.91 ±2.51	2.51	0.01 ±0.00	116.40	98.27 ±4.71	1.36
		27.26	116.06 ±2.32	2.32		58.20	94.92 ±8.92	2.10
		5.43	62.01 ±35.51	33.51		11.64	98.23 ±7.93	6.18
Geranic acid	0.79 ±0.09	116.44	70.00 ±12.90	12.90	8.14 ±3.99	2301.89	95.89 ±1.28	1.89
		58.22	75.63 ±7.16	7.16		1150.95	85.33 ±3.14	10.27
		11.64	80.23 ±5.68	2.16		230.19	83.19 ±2.90	18.82
**C_{13}-Norisoprenoids**								
β-Damascenone	0.00	0.23	85.44 ±5.66	5.66	0.00	1.01	88.16 ±3.64	4.40
		0.11	88.32 ±6.89	6.89		0.50	88.47 ±1.89	2.80
		0.02	84.23 ±5.50	5.50		0.10	89.12 ±2.76	2.95
Geranyl acetone	0.07 ±0.01	2.00	66.70 ±6.91	6.91	0.04 ±0.01	1.90	105.25 ±11.00	2.46
		1.00	74.45 ±4.01	4.01		0.95	103.83 ±17.64	3.83
		0.10	120.53 ±21.66	21.66		0.19	104.31 ±19.13	5.23
β-Ionone	0.04 ±0.01	1.96	93.51 ±1.00	1.00	0.01 ±0.01	2.11	78.29 ±5.27	7.59
		0.98	86.49 ±0.63	0.63		1.05	83.32 ±9.50	6.74
		0.20	84.80 ±0.52	0.52		0.21	112.30 ±26.00	3.73

Table S6. Recovery (%) and reproducibility (relative standard deviation, r.s.d.) (%) of the target skin and pulp juice volatile compounds in table grape.
